# Global profiling of protein complex dynamics with an experimental library of protein interaction markers

**DOI:** 10.1038/s41587-024-02432-8

**Published:** 2024-10-16

**Authors:** Christian Dörig, Cathy Marulli, Thomas Peskett, Norbert Volkmar, Lorenzo Pantolini, Gabriel Studer, Camilla Paleari, Fabian Frommelt, Torsten Schwede, Natalie de Souza, Yves Barral, Paola Picotti

**Affiliations:** 1 Institute of Molecular Systems Biology, Department of Biology, ETH Zurich, Zurich, Switzerland; 2 Institute of Biochemistry, Department of Biology, ETH Zurich, Zurich, Switzerland; 3 Biozentrum, University of Basel, Basel, Switzerland; 4 SIB Swiss Institute of Bioinformatics, Computational Structural Biology, Basel, Switzerland

**Keywords:** Dynamic networks, Structural biology, Proteome informatics

## Abstract

Methods to systematically monitor protein complex dynamics are needed. We introduce serial ultrafiltration combined with limited proteolysis-coupled mass spectrometry (FLiP–MS), a structural proteomics workflow that generates a library of peptide markers specific to changes in PPIs by probing differences in protease susceptibility between complex-bound and monomeric forms of proteins. The library includes markers mapping to protein-binding interfaces and markers reporting on structural changes that accompany PPI changes. Integrating the marker library with LiP–MS data allows for global profiling of protein–protein interactions (PPIs) from unfractionated lysates. We apply FLiP–MS to *Saccharomyces cerevisiae* and probe changes in protein complex dynamics after DNA replication stress, identifying links between Spt-Ada-Gcn5 acetyltransferase activity and the assembly state of several complexes. FLiP–MS enables protein complex dynamics to be probed on any perturbation, proteome-wide, at high throughput, with peptide-level structural resolution and informing on occupancy of binding interfaces, thus providing both global and molecular views of a system under study.

## Abstract (web summary)

FLiP–MS uses a library of protein–protein interaction markers to understand protein complex dynamics.

## Main

Most biological processes are executed by proteins, often as part of dynamic protein complexes that assemble, disassemble or rearrange in response to environmental changes or internal cues. The composition and structure of protein complexes affect protein function, and the state of a cell is dictated by an interconnected network of protein–protein interactions (PPIs)^1^. Alterations in PPI networks can cause disease^2–10^, and disease-associated mutations are enriched at protein-binding interfaces (PBIs)^11^. Disrupting^12^ and stabilizing^13^ PPIs is gaining increasing pharmacological relevance^14^. Further, profiling protein complexes and their dynamics is key to understanding and predicting biological systems.

Affinity-purification mass spectrometry (AP–MS) and chromatographic fractionation mass spectrometry (CF–MS) have revealed a wealth of PPIs. For instance, an AP–MS study reported over 118,000 pairwise interactions between more than 14,500 human proteins^15^. A recent CF–MS study found 612 putative *Caenorhabditis elegans* protein complexes covering one-fourth of the worm’s proteome^16^. But current approaches probing system-wide interactomes lack sufficient proteome coverage and/or are labor- and time-consuming for the analysis of multiple conditions. A CF–MS comparison of the interactome of five species required roughly 9 months of MS measurement time^17^ and is not sufficiently scalable for routine studies of interactome dynamics in response to perturbation. In addition, CF–MS approaches do not allow identification of PBIs^18^. Crosslinking coupled to mass spectrometry (XL–MS) can identify PBIs and is increasingly applied to complex lysates. Recent work has identified hundreds of interprotein crosslinks in yeast, *Escherichia coli*, mouse synapses and human cytoplasm^19–22^, but the relatively low abundance of cross-linked peptides leads to low proteome coverage or requires fractionation of samples, which decreases throughput. There is an unmet need for approaches that monitor protein complexes systematically, dynamically, at high throughput and with sufficient structural resolution to identify PBIs.

Information about PBI location could guide mutation studies or small-molecule design to modulate the assembly of specific protein complexes, probing their functions and links to phenotypes^4,11^. It would support docking and structure prediction studies of complexes for which no structure is available and help predict the effects of mutations on complex stability^23,24^. Information about altered PBI occupancy in disease could also guide the identification of new drug targets in the affected protein networks^25^.

Structural proteomics tools such as limited proteolysis-coupled mass spectrometry (LiP–MS), previously developed in our laboratory^26,27^, can capture protease susceptibility changes due to PPIs with peptide-level resolution, but LiP–MS also detects numerous other molecular events. These may include allosteric changes, posttranslational modifications, conformational changes associated with altered enzyme activity and small-molecule protein binding^27^. Changes in PPIs are only one of many types of structural alteration simultaneously captured by LiP–MS and this information cannot be disentangled in the absence of additional data.

We now introduce serial ultrafiltration combined with LiP–MS (FLiP–MS), a new structural systems biology workflow based on LiP–MS that pinpoints protease susceptibility changes due to altered PPIs, enabling fast, global analyses of protein complex dynamics. FLiP–MS differs from LiP–MS in that it prioritizes the identification of quaternary structural changes, that is, those due to changes in PPIs. FLiP–MS relies on the identification of a library of peptide markers that report on changes in protein complex assembly state. These include peptides directly located at PBIs and peptides involved in protein structural changes that accompany PPIs. These markers can then be monitored in subsequent LiP–MS analyses to globally detect changes in PPIs across conditions. The approach is modular, with multiple informative stopping points depending on the biological question being tackled.

We applied FLiP–MS to the *Saccharomyces cerevisiae* proteome, identified PPI markers for 1,086 proteins and evaluated candidate markers using experimental and AlphaFold-predicted structures, functional enrichment analysis, and information on mutations known to affect PPIs. We then applied these PPI markers to track global changes in PPIs in yeast under hydroxyurea (HU)-induced DNA replication stress. We recapitulated several known PPI alterations, detected new links between acetylation and protein complex assembly states of Spt-Ada-Gcn5 (SAGA) acetyltransferase targets, and discovered a role for SAGA activity in the formation of P-bodies, dynamic biological condensates of messenger RNAs (mRNAs) and proteins that form via phase separation and regulate mRNA fate. Combined structural and systems-level analysis with FLiP–MS should enable dynamic studies of protein complex rearrangement under any perturbation of interest.

## Results

### FLiP–MS globally probes protein complex dynamics

**Fig. 1:**
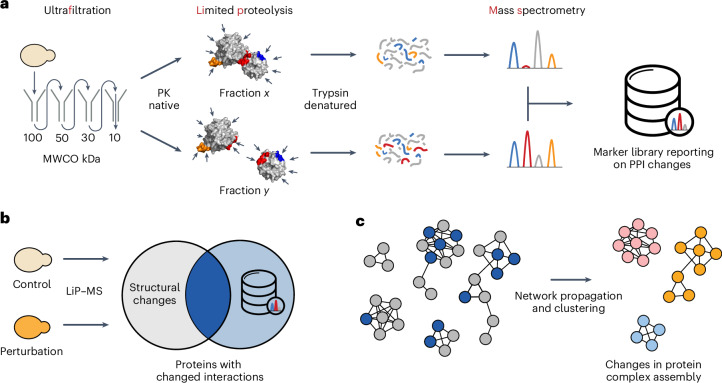
The modular FLiP–MS approach. **a**, Generating the FLiP marker library. A lysate is subjected to serial ultrafiltration to separate large protein complexes from their monomeric subunits. The fractions are separately subjected to limited proteolysis under native conditions, during which the sequence-unspecific protease PK cleaves surface-accessible residues. Regions at PBIs (red) should be accessible to PK cleavage in the monomeric form but sterically shielded in the complex-bound form, thus LiP should generate differential cleavage patterns. Many surface-accessible regions not located at the PBI (blue) should be equally accessible in both monomeric and complex-bound forms, thus LiP should generate the same cleavage patterns. Regions of proteins not at the interface but that also change protease susceptibility in the two assembly states (orange) will also generate different cleavage patterns. After the LiP step, protein fragments from each fraction are denatured, digested with trypsin and analyzed by mass spectrometry. Differential PK cleavage between the monomeric and the complex-bound forms of a protein is reflected in differential abundances of peptides between fractions. Consequently, differential peptide abundance analysis between fractions identifies markers reporting on PPI changes on a proteome-wide scale and enriches for PBIs (PDB ID 4DSS is shown as an example structure). Figure adapted from ref. ^26^. **b**, Combining the FLiP library with LiP–MS. Conventional LiP–MS experiments report on global structural protein alterations between two conditions. The overlap with the FLiP marker library identifies which structural alterations may represent changes in PPIs. **c**, Network analysis for a global view of interactome dynamics. The identified changes in PPIs are projected on publicly available interaction networks, followed by network propagation and clustering to identify regions of the interactome that change on a given perturbation.

We sought to develop a high-throughput approach to systematically analyze protein interactome dynamics across conditions from unfractionated cell lysates that also provides structural information about PPIs. Briefly, we combined serial ultrafiltration with LiP–MS to identify peptides that change protease accessibility when different assembly states of a protein are compared, on a proteome-wide scale in native cell extracts (Fig. 1a). This generated a new resource of peptide markers that report on PPI alterations, which we term the FLiP marker library. The marker set is enriched in peptides directly mapping to PBIs and may contain also peptides mapping to regions involved in structural changes that accompany protein complex formation or dissolution. This library is the first output of our modular approach and could be used as a standalone resource to help design mutations or drugs to interfere with PPIs, for functional characterization of protein complexes or as training or evaluation data for multimeric protein structure predictions. For FLiP–MS, the marker library is subsequently integrated with any LiP–MS analysis to enable global monitoring of proteins changing their PPIs under conditions of interest (Fig. 1b). Whereas LiP–MS generates a global picture of protease susceptibility changes on a perturbation of interest, which may be due to numerous classes of molecular events (for example, posttranslational modifications, allosteric changes, interactions), FLiP–MS reports on protease susceptibility changes that are likely to reflect changes in PPIs. The pipeline may also be halted after this step since the resulting data allow the generation of hypotheses on the involvement of selected PPIs in a cellular response of interest and the identification of potential deregulated interfaces. As a further optional step, integrating PPI changes with known protein interaction networks identifies network regions rich in interaction changes (Fig. 1c), providing a systems-level and protein complex-centric view of interactome dynamics during the cellular response to a perturbation.

### A library of PPI markers

Our approach to experimentally identify changing protein complexes on a global scale is based on the rationale that PBIs are known to show differences in protease susceptibility^28,29^ between the complex-bound form, where the PBI should be sterically blocked by the interaction partners, and the monomeric form, where the PBI should be protease-accessible. Thus, identifying changes in protease susceptibility by LiP–MS between the complex-bound and monomeric form of a protein should pinpoint PBIs (Fig. 1a). It must be noted, however, that different assembly states of a protein may be conformationally different also in regions that are not directly at the interface. Some such regions may also show differential protease susceptibility between the monomeric and the complex-bound form.

**Fig. 2:**
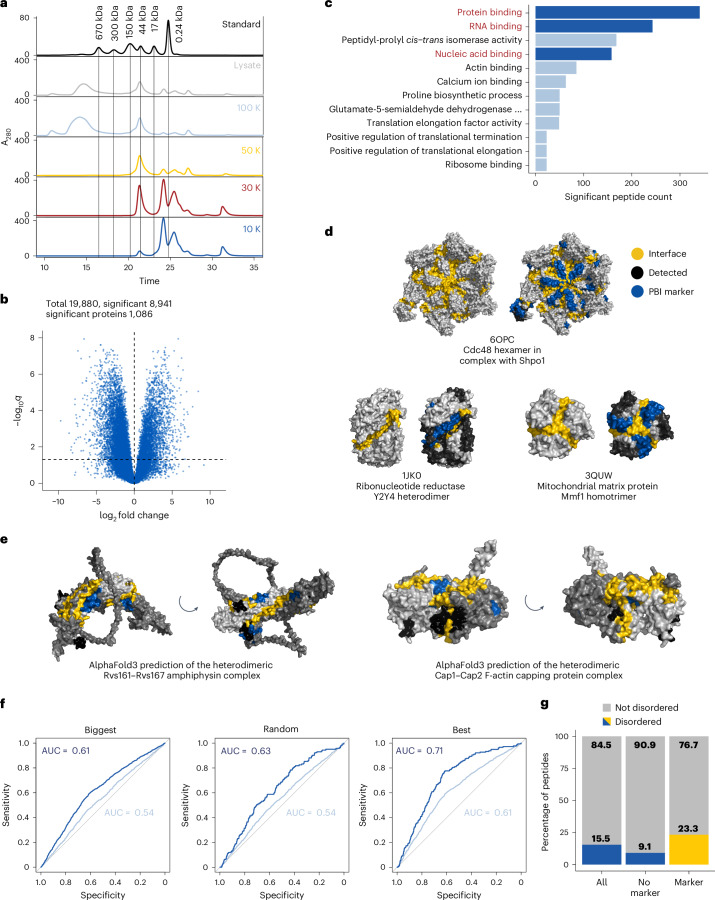
Characterization of the FLiP–MS library. **a**, SEC ultraviolet absorption traces of 100 μg of protein from the lysate, 100-K, 50-K, 30-K and 10-K fraction. **b**, Volcano plot showing the differential abundance of peptides between fractions for all peptides identified in at least two filter fractions. Peptide intensities were corrected for protein abundances. If a peptide was identified in more than two fractions, the represented fold change corresponds to the maximal fold change between any fractions. *P* values were determined by an ANOVA (one-way) and corrected for multiple testing (Benjamini–Hochberg). We tested a total of 19,880 peptides, of which 8,941 peptides from 1,086 proteins significantly changed abundance between filter fractions (adjusted *P* < 0.05). **c**, GO-enrichment analysis of FLiP markers based on InterPro domain annotations using Fisher’s exact test (one-sided, *P* < 0.05). **d**, Projection of PBIs and the FLiP–MS dataset on PDB structures of three exemplary protein complexes. The PBI (yellow) is mapped to both left and right structures for each pair. The FLiP marker peptides (blue) and detected but unchanging peptides (black) are shown at right for each pair. **e**, Projection of PBIs and the FLiP–MS dataset on AlphaFold3-predicted structures of the Rvs161–Rvs167 and the Cap1–Cap2 complex. The color code is the same as in **d**. **f**, ROC curves assessing mapping of marker peptides to known PBIs. All peptides from the FLiP dataset with available multimeric PDB structures (*n* = 364–373 multimeric structures) are included in the analysis. If multiple PDB structures are available for a given protein, we chose the PDB structure with the most subunits (Biggest), a random PDB structure (Random) or the PDB structure that best matched our data (Best). For all analyses, a peptide was defined as at the interface if its average distance to the interface was <2.6 Å (light blue) or <0.3 Å (dark blue). The gray line represents a random classifier. **g**, Percentage of disordered peptides in the complete, nonsignificant and significant FLiP datasets.

To separate large protein complexes from their respective monomeric subunits, we applied serial ultrafiltration to a yeast lysate prepared under native conditions. Given the importance of RNA-binding proteins in cellular organization, for instance in the formation of biomolecular condensates, we designed the workflow to also capture changes in RNA-dependent protein complexes and in RNA–protein complexes. We incubated lysates with RNases before size separation leading to a destabilization but not a complete disassembly of RNA-dependent complexes (Methods). The lysate was loaded onto a 100-kDa molecular weight cutoff (MWCO) filter, and the flow-through was sequentially loaded onto 50-, 30- and 10-kDa MWCO filters resulting in four fractions. Analysis of the fractions under native conditions showed that they comprised proteins and protein assemblies of progressively decreasing molecular weight based on both size-exclusion chromatography (SEC) (Fig. 2a) and Native PAGE (Supplementary Fig. 1a,b). Notably, each fraction showed a unique size distribution in SEC relative to all other fractions. The 100-K fraction was uniquely enriched for large protein assemblies (>670 kDa), the 10-K fraction was uniquely depleted of proteins larger than 17 kDa and the two intermediate fractions differed from all other fractions in their size distributions. A complete separation between fractions is not needed for our approach. The key requirement is that there is an enrichment of different molecular weight forms in different fractions, as we observe.

Quantitative proteomic analysis of the fractions in quadruplicate filtration experiments identified between 1,763 and 2,047 proteins in the individual fractions and a total of 2,386 proteins (Supplementary Fig. 1c). Although many (1,466) proteins were shared between all four filter fractions (Supplementary Fig. 1d), the relative levels changed. Out of the 2,096 proteins found in two or more fractions, 1,296 proteins changed their abundance significantly between fractions (Supplementary Fig. 1e, adjusted *P* < 0.05, |log_2_ fold change| > 0.5), again indicating that we enrich for different sets of proteins in different fractions; this was confirmed in a clustering analysis (Supplementary Fig. 1f).

Analysis of the median molecular weight of individual proteins in each fraction showed an increase in molecular weight between the 10-K, 30-K and 50-K fractions (from 18.5 to 23.5 to 31.5 kDa), as expected (Supplementary Fig. 1g). The median molecular weight in the 100-K fraction of 27.5 kDa was unexpectedly lower than that in the 50-K fraction, suggesting that the 100-K fraction is enriched in large protein assemblies formed by low molecular weight monomers. Note that most of the proteins in the full yeast lysates have a molecular weight <50 kDa (Supplementary Fig. 1h).

We subjected all four fractions to the LiP–MS workflow to identify changes in protease susceptibility between putative complex-bound and monomeric forms of a protein, for proteins found in multiple molecular weight fractions. During the limited proteolysis step, proteinase K (PK), a sequence-unspecific protease, is applied to proteins in a native state for a short period. This allows PK to cleave surface-accessible regions of the protein^26,30,31^. Regions at PBIs should be accessible to PK cleavage in the monomeric form but sterically shielded in the complex-bound form, thus LiP should generate differential cleavage patterns. Many surface-accessible regions not located at the PBI should be equally accessible in both monomeric and complex-bound forms, thus LiP should generate the same cleavage patterns. Some regions of proteins not at the interface may undergo allosteric changes on PPI alterations and therefore also change protease susceptibility in the two assembly states; these will also generate different cleavage patterns for monomeric and complex-bound forms. Next, the protein fragments were denatured and digested with the sequence-specific protease trypsin, and the resulting peptides quantified by label-free mass spectrometry using data-independent acquisition (DIA). Differences in surface accessibility of structural states of a protein, such as between a complex-bound and monomeric form, can be detected by distinct proteolytic cleavage patterns (Fig. 1a).

LiP–MS of each filter fraction identified between 18,304 and 24,445 peptides, for a total of 35,951 peptides from 2,039 proteins across all fractions (Supplementary Fig. 2a,b). These include both semitryptic peptides (that is, those typically formed by one PK and one trypsin cleavage event) and fully tryptic peptides (that is, those formed by two trypsin cleavages). As expected for a filtration series separating proteins based on their molecular weight, only one-fifth of the peptides (7,091) were identified consistently in all four filter fractions (Supplementary Fig. 2c). The percentage of semitryptic peptides, a typical control for the efficiency of the PK digestion step^30^ was comparable across fractions, indicating that differences in proteolytic cleavage patterns were not due to different PK activity in the different fractions (Supplementary Fig. 2d). After normalization for protein abundance variation across fractions, we statistically compared the intensities of LiP peptides identified in at least two filter fractions for each protein. Of 19,880 such peptides, 8,914 significantly changed in abundance across fractions (analysis of variance, ANOVA, adjusted *P* < 0.05; Fig. 2b), with the strongest changes occurring between the 100-K and smaller fractions (Supplementary Fig. 2e). We further grouped overlapping peptides and defined a final set of 6,441 FLiP markers mapping to unique protein regions. These serve as PPI markers, including putative PBIs, in 1,086 proteins (Supplementary Table 1).

Cleavage events or other features that cause a protein to fractionate in an unexpected way can be biologically meaningful but can also reflect technical noise. To take this into consideration, we classified markers as high confidence if the protein was found in no smaller than the expected filter fraction; this holds true for 85% (5,481 markers) of the FLiP marker library. Additionally, neither contact with filter membranes nor processing time led to structural changes that could be detected by LiP–MS (Supplementary Fig. 2f,g), indicating that the filtration process does not induce structural changes. Also, 90% of the markers span 57.5% of the most abundant yeast proteins and are thus not limited to highly abundant proteins^32^ (Supplementary Fig. 3a). Further, markers mapped to complexes with a broad range of stoichiometries (Supplementary Fig. 3b) and dissociation energies (Supplementary Fig. 3c), consistent with interactomes characterized by affinity-purification techniques^33,34^. As for all proteomics-based methods, protein sequence coverage in the FLiP–MS dataset is dependent on protein abundances (Supplementary Fig. 3d). Also, there is a slight bias to detect FLiP markers for proteins with medium to high abundance, which is expected since the resulting higher sequence coverage will increase the likelihood of detecting any structural change (Supplementary Fig. 3e). No difference in these trends was observed between filter fractions (Supplementary Fig. 3e).

Since we expected the FLiP markers to be enriched at PBIs, we assessed the validity of this hypothesis with orthogonal analyses. To probe the functional annotation of FLiP markers, we tested for enrichment based on domain-level gene ontology (GO) information (InterPro^35^). Among enriched terms, ‘protein binding’ was the most abundant, followed by ‘RNA binding’ (Fisher exact test, adjusted *P* < 0.01; Fig. 2c), consistent with markers being enriched at protein-binding or nucleic-acid-binding interfaces. Note that this analysis probes enrichment within specific protein regions (the FLiP markers) and not in the whole protein since InterPro protein-binding domains are <120 amino acids (aa) for most domains and <50 aa for half of the domains.

We next asked whether marker peptides are close to binding interfaces visible in high-resolution structures of known protein complexes. We used a rolling ball algorithm^36^ to define PBIs and mapped marker peptides as well as other detected but nonchanging peptides to all available multimeric Protein Data Bank (PDB) structures (Fig. 2d). In examples ranging from heterodimers (PDB 1JK0, ref. ^37^) to homotrimers (PDB 3QUW, ref. ^38^) and complex hexameric structures (PDB 6OPC, ref. ^39^), the FLiP markers pinpoint the binding interfaces while other detected peptides that are not FLiP markers are located further away (Fig. 2d). In total, 1,078 FLiP markers from 275 proteins map to known PBIs, defined either based on a distance <2.6 Å to a PBI in a multimeric PDB structure or by the peptide being located in an InterPro domain annotated with the GO-term ‘protein binding’ (Supplementary Table 1). The FLiP markers map to 381 protein from 295 complexes in the Complex Portal database^40^. Of these, only 148 proteins from 153 complexes have previous evidence for a protein-binding interface. Thus, many of the FLiP markers could report on binding interfaces of previously uncharacterized protein complexes. Finally, we predicted the structure of the heterodimeric Rvs161–Rvs167 amphiphysin complex with AlphaFold3 and observed that FLiP markers of both binding partners mapped to the predicted PBI while nonmarker peptides mapped further away (Fig. 2e, left). In the case of the AlphaFold3-predicted heterodimeric structure of the Cap1–Cap2 F-actin capping protein complex, the FLiP peptide of one binding partner is at the predicted interface while the other one is located further away (Fig. 2e, right).

Global extension of this analysis was challenging, mainly due to the lack of a good ground truth dataset of PBIs across the proteome. Many PPIs remain uncharacterized^41,42^ and would not be represented in the PDB, the most comprehensive database of protein structures to date. Conversely, not all protein complexes in the PDB are expected to be present in our lysate since some complexes may need specific conditions to form. Some structures in the PDB were generated under conditions not relevant to our dataset including mutations, additional binding partners or nonphysiological conditions. Nevertheless, we mapped the sequences of all peptides of the FLiP–MS dataset (marker and nonmarker peptides) to multimeric PDB structures.

For many proteins, several multimeric structures are available (Supplementary Fig. 3f). To avoid overrepresentation of a protein with multiple structures, we had to choose one of them for mapping the peptides. To select a single structure for each protein in an unbiased way, we chose the PDB structure with either the highest number of subunits (biggest), reasoning that these complexes contain the highest number of PBIs, or at random (random), both of which should be unbiased sets. In addition, we also generated a best-case ground truth set in which we selected the PDB structure best matching our data (best), reasoning that since the PDB contains structures that may not be relevant to our experiment (for example, drug-bound, mutated or truncated forms, or different conditions such as pH), this best-case dataset would be most likely to select structures that were solved under the appropriate conditions.

For each ground truth set, we calculated the average minimal distance between all atoms of a peptide and all atoms of the PBI in the corresponding PDB structure to capture how close the peptide is to a known interface in three-dimensional space. We defined any peptide with distance <2.6 Å to be located at the PBI, or more stringently required the whole peptide to be located at the interface (distance <0.3 Å). Depending on the ground truth set (biggest, random or best), we mapped between 6,428 and 6,592 peptides to 373–394 PDB structures by sequence. All peptides that could not be mapped to a structure were discarded from the analysis. Of the mapped peptides, 1,349–1,358 peptides (distance <2.6 Å) or 143–179 peptides (distance <0.3 Å) were at an interface (Supplementary Table 2). A receiver operating characteristic (ROC) analysis (Methods) showed that FLiP markers are more likely to map to PBIs than expected by chance, with performance improving for the more stringent (distance <0.3 Å) analysis, and for the best ground truth dataset as expected (Fig. 2f). Requiring a 100% sequence overlap for a peptide to be located at the interface gives similar results to setting the cutoff to 0.3 Å with all three ground truth datasets (Supplementary Fig. 3g). These data show that the FLiP marker library is enriched for peptides at PBIs.

It is expected that the FLiP marker library includes peptides that do not map to PBIs. The pipeline will also capture structural rearrangements at regions other than the PBI that accompany the formation or dissociation of a protein complex, including conformational changes or binding of small molecules needed for complex formation^43–45^. However, some of the false positives are likely to arise because the PDB constitutes an imperfect ground truth. To better understand the nature of these false positives, that is, the 72% of FLiP marker peptides that did not map to a known PBI in the PDB (best), we used domain-based GO enrichment to ask to which types of domain these peptides map. We performed an InterPro domain-based GO-enrichment analysis comparing against mapped but nonchanging peptides as background. False-positive peptides in proteins with only false positives (422 peptides from 114 proteins) were significantly enriched in protein-binding domains (*n* > 60) and also RNA-binding domains (*n* > 10, Supplementary Fig. 3h), indicating that these peptides may not be false positives and that the PDB-based analysis may underestimate the performance of our method. In contrast, in proteins that have both false and true positives (1,635 and 533, respectively, from 130 proteins), false-positive peptides were enriched in GTP and ATP binding (*n* > 100), metal ion binding (*n* > 50) domains and RNA-binding domains (*n* > 50) (Supplementary Fig. 3i). Since the number of changing markers per protein in this group is high, this set of proteins may undergo more substantial structural rearrangements during complex formation. In general, proteins with many changing FLiP markers are likely to include peptides that also map to protein regions other than the PBI. We note that FLiP markers are enriched in disordered regions (*P* = 2.9 × 10^−13^, one-sided Fisher’s exact test) (Fig. 2g), agreeing with reports that many PBIs are disordered^46^.

As a last assessment of the FLiP library, we asked whether it is enriched for mutations that destabilize protein–protein or protein–RNA interactions, which typically involve PBIs^3,11,47,48^, using the manually curated IMEx collection^49,50^. Here, 46 mutations were in actin, and we excluded them from the analysis to avoid biases. In the remaining 118 proteins with at least one FLiP marker, the markers overlapped with 82.5% of all detected mutation sites (63 detected mutations out of 285 total mutations). A random draw of the same number of mutations (285 mutations, sampled ten times) from the same set of proteins showed a significantly lower overlap with FLiP markers (78.6%, *P* < 0.0008). The proportion of FLiP markers mapping to mutation sites increased to 86.1% (83.1% for randomly placed mutations, *P* < 0.002) when allowing for mutations within ten amino acids from the marker peptide. Despite the high overlap of FLiP markers with randomly placed mutations, these data show an enrichment of the markers at or close to mutation sites that disrupt protein–protein and protein–RNA interactions. In the case of actin, the 46 reported mutations cover the entire sequence while the FLiP markers mapped mostly to evolutionarily conserved regions (Supplementary Fig. 3j), consistent with conservation of key PBIs. Taken together, multiple lines of evidence, including GO-enrichment analysis, mapping to PDB structures, and mutational analysis indicate that markers identified by FLiP–MS are enriched for PBIs.

### FLiP–MS tracks known PPI changes on DNA replication stress

The FLiP marker library can be applied to identify changes in PPIs on perturbation from unfractionated cell extracts. Classical LiP–MS analyses detect structurally altered proteins based on distinct proteolytic patterns, but these reflect not only PPI changes but also protein-small-molecule binding, posttranslational modifications or conformational changes^27^. To disentangle this information, we reasoned that changing LiP peptides that correspond to FLiP markers should specifically report on changes in PPIs between conditions (Fig. 1b). We tested this by studying HU-induced DNA replication stress in yeast^51–54^. HU is a drug used in cancer chemotherapy^55^ and in the treatment of other diseases^56^. It inhibits ribonucleotide reductase^57^ and thereby depletes the cell of deoxynucleoside triphosphates, resulting in DNA replication stress^58^. In response to HU treatment, the chromatin remodeling complexes INO80 and RSC are recruited to stalled replication forks^59–62^, and P-bodies form in the cytoplasm by liquid–liquid phase separation to regulate mRNA fate^63–65^. Thus, this treatment is known to induce the remodeling of PPIs with different biochemical features.

**Fig. 3:**
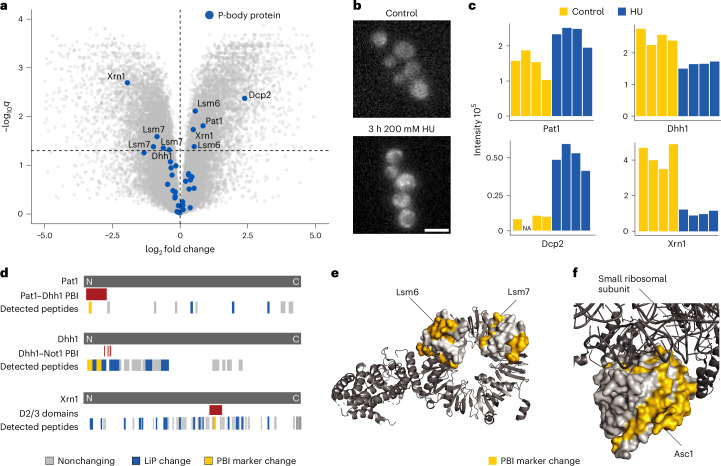
FLiP–MS captures known PBI changes in P-body protein complexes on DNA replication stress. **a**, Volcano plot showing differential abundance analysis of peptides from LiP–MS on HU-induced DNA replication stress. Peptide abundances were corrected for protein abundance changes between conditions. *P* values were calculated by an ANOVA (one-way) and corrected for multiple testing (Benjamini–Hochberg). The dotted horizontal line indicates an adjusted *P* value of 0.05. Blue dots represent FLiP markers of P-body proteins. **b**, Fluorescence micrographs showing yeast cells expressing the P-body marker Dcp2 tagged with GFP, with and without exposure to HU. Scale bar, 5 μm. **c**, Mass spectrometry signal intensities of FLiP markers for selected P-body proteins in control and under HU stress; four replicates are shown. **d**, The schematic shows selected P-body proteins with the location of several features marked along the protein sequence. Shown are known PBIs (red), FLiP markers (yellow), structural changes on HU stress identified by LiP–MS (blue) and detected but nonchanging peptides (gray). **e**, Structure of the Pat1–Lsm1-7 complex (PDB 4C8Q, ref. ^71^). The FLiP markers significantly changing on HU stress for Lsm5, Lsm6 and Lsm7 are shown in yellow. **f**, Structure of Asc1 (light gray) in complex with the small ribosomal subunit (dark gray) (PDB 6FAI, ref. ^74^). The FLiP markers significantly changing on HU stress are shown in yellow.

We grew yeast under HU stress for 2 hours and assessed global structural changes compared to yeast grown without stress. We performed LiP–MS of whole yeast cell lysates using label-free mass spectrometry based on DIA^27^ and then intersected the list of altered peptides in the LiP–MS dataset with our FLiP library. Out of 3,392 detected proteins, only 533 changed abundance (ANOVA, |log_2_ fold change| > 0.5, adjusted *P* < 0.05; Supplementary Fig. 4a and Supplementary Table 4). After correcting for protein abundance changes, we detected significant structural alterations on HU treatment for 1,814 proteins based on 10,368 altered peptides out of the 34,672 identified peptides (ANOVA, adjusted *P* < 0.05; Fig. 3a, Supplementary Fig. 4b and Supplementary Table 4). Out of these 10,368 peptides indicating structural changes, 3,085 peptides from 586 proteins correspond to FLiP markers (at least 50% of the peptide overlapping with marker, 2,824 high confidence and 261 low confidence markers) and thus report on potential changes in PPIs on HU treatment. The FLiP-shortlisted hits correspond to protein regions enriched for protein-binding and RNA-binding domains, whereas the full set of LiP–MS hits do not show this enrichment (Supplementary Fig. 4c,d). FLiP markers were also slightly but significantly nearer the solvent accessible protein surface than the remaining LiP peptides (Supplementary Fig. 4e).

To validate that intersecting the FLiP library with these LiP–MS data identified changes in PBI occupancies expected to occur under DNA replication stress, we asked whether the approach identified changes reflecting P-body formation. Notably, the FLiP library includes markers for eight P-body proteins despite P-bodies not being microscopically visible under the growth conditions used for library generation, indicating the presence of higher order assemblies even at steady state. Having confirmed that P-bodies formed under HU stress using a yeast strain expressing green fluorescent protein (GFP)-tagged Dcp2 (Fig. 3b), a known P-body marker, we asked whether FLiP markers changed for the 13 most abundant proteins in P-bodies^66^ (Dcp2, Edc3, Pat1, Xrn1, Lsm1-7, Upf1, Dhh1). Out of the eight detected P-body proteins with FLiP markers, we found six proteins with changing markers (Pat1, Lsm6, Lsm7, Dhh1, Dcp2 and Xrn1, Fig. 3a). The marker peptides showed clear differences in mass spectrometry signal intensities under HU stress compared to control conditions (Fig. 3c), reflecting a change in accessibility of the corresponding protein regions.

Several of the changing FLiP markers are consistent with PPIs known to regulate P-body formation and mapped to known binding interfaces of the P-body interactome. One such marker mapped to the mRNA-decapping factor Pat1, directly at its binding interface to the DEAD-box protein Dhh1; an interaction known to be crucial for P-body formation^67,68^ (Fig. 3d). To efficiently bind RNA, Pat1 interacts with the Lsm1-7 complex^69–71^, for which we found changing marker peptides for Lsm6 and -7, in all cases mapping to binding interfaces in the heptametric ring structure (Fig. 3e, PDB 4C8Q). Further, we identified two changing FLiP markers for Dhh1, located next to the Not1 binding interface, an interaction known to inhibit P-body formation^68,72^ (Fig. 3d). We also detected several structural changes for Not1, but our FLiP marker library does not cover this protein because its large size (240 kDa) exceeds the separation range of our pipeline. We identified one changing marker for the 5′–3′ exonuclease Xrn1, located at the D2/D3 domain suggested to be involved in protein binding^73^ (Fig. 3d). Last, we analyzed the G-protein subunit Asc1, known to be required for P-body formation under HU stress and to bind the small ribosomal subunit^65^. We observed seven significantly changing FLiP markers for Asc1 on HU treatment, most of which were in close proximity to the binding interface with the small ribosomal subunit (Fig. 3f, PDB 6FAI, ref. ^74^).

Overall, our data show that integrating the FLiP marker library generated with FLiP–MS into a global structural LiP–MS analysis of cells under DNA replication stress detected known changes in PPIs and PBI occupancy of several specific complexes involved in P-body formation, a known component of the cellular response. This suggests that the FLiP marker library could be used for discovery of previously unknown protein complex dynamics.

### Global interactome dynamics under DNA replication stress

**Fig. 4:**
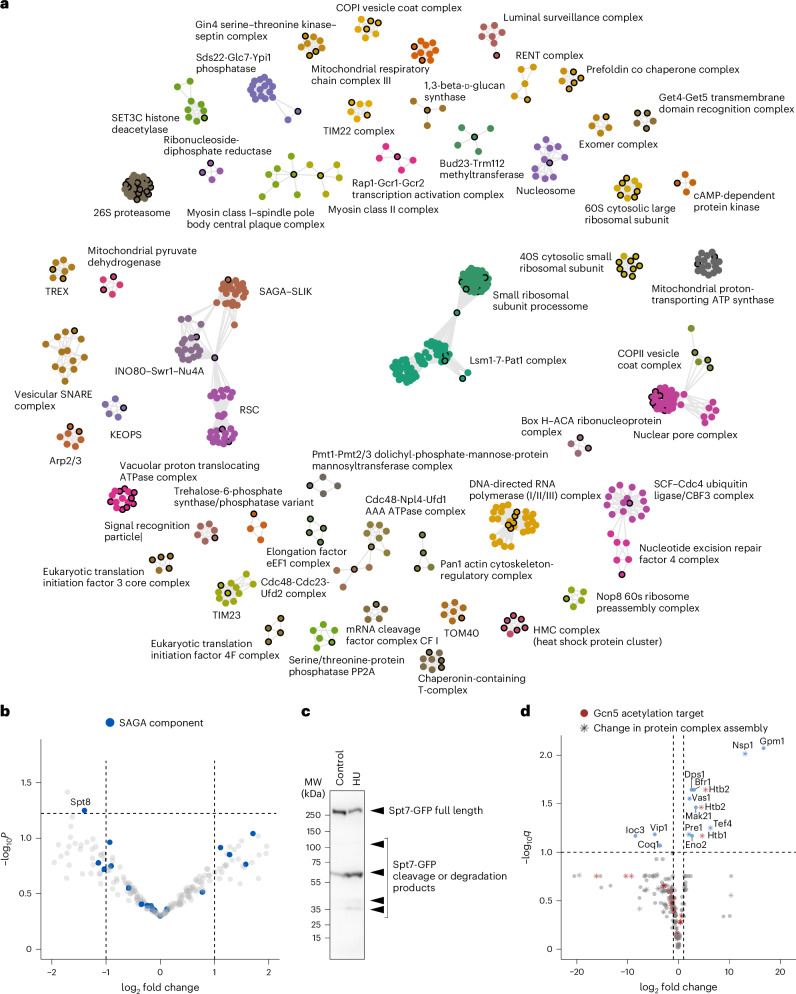
Global PPI dynamics under DNA replication stress reveals a role for the SAGA complex. **a**, Protein complexes likely to change assembly state under DNA replication stress. Proteins with changing FLiP markers are indicated by a black circle. **b**, Differential abundance analysis of proteins coimmuno-precipitated with Ada3-FLAG in anti-FLAG AP–MS under HU stress compared to control. *P* values were calculated by a Student *t*-test (two-sided) for the ratio of prey in the pull-down versus empty control. A negative log_2_ fold change indicates less interaction of the prey under HU stress. SAGA component proteins are indicated in blue. **c**, Western blot of C-terminally GFP-tagged Spt7 under control and HU stress (single replicate; MW, molecular weight). **d**, Volcano plot showing differential abundance of acetylated peptides between yeast grown under HU stress and control conditions. A positive fold change corresponds to higher acetylation under HU stress. *P* values were determined with an ANOVA test (one-way) and corrected for multiple testing (Benjamini–Hochberg). The protein corresponding to each peptide is indicated. Known acetylation targets are indicated in red, proteins belonging to a protein complex suggested to undergo changes in assembly state are indicated with a star. Source data

Integrating the FLiP PPI marker library into a LiP–MS experiment identifies proteins that may change their interactions but does not contain information about the specific binding partners causing the change. To identify these binding partners, we made use of previous knowledge. We reasoned that simultaneous changes of FLiP markers of multiple proteins that are known to form a protein complex may indicate assembly or disassembly of the complex. We therefore projected the markers that changed on DNA replication stress onto a PPI network from the Complex Portal database^40,75,76^. Out of the 586 proteins with changing FLiP markers, only 206 are part of this manually curated database. We used network propagation to identify proteins closely connected to our marker hits and clustered proteins in the resulting network based on density of interactions, annotated each cluster as a particular protein complex (Methods), filtered for proteins with at least four subunits and interrogated which protein complexes change (defined as ≥1 changing FLiP marker) between conditions (Supplementary Fig. 5a,b). This analysis identified 56 protein complexes with between one and 13 changing FLiP markers on HU stress (Fig. 4a and Supplementary Table 5), out of which 52 complexes had high confidence markers. For this exploratory analysis, we defined a complex as changing if it had even a single changing FLiP marker, since even the assembly or disassembly of a single protein subunit could be biologically impactful. We note, however, that the higher the number of detected FLiP marker changes per complex, the higher the likelihood that this complex undergoes complete assembly or disassembly under DNA replication stress.

Previous genetic and other orthogonal data suggest that our results are functionally relevant. We found multiple interconnected altered protein complexes, namely SAGA, INO80–Swr1–Nu4A and RSC that have been previously implicated in the response to DNA replication stress. Mutations in SAGA render cells more sensitive to HU^77^, while the INO80 (ref. ^59,60^), Swr1 (ref. ^78^), Nu4A (ref. ^79^) and RSC^61,62^ complexes are recruited to stalled replication forks in response to DNA replication stress or damage. Our analysis also identified the nucleotide excision repair factor 4 complex involved in DNA damage repair^80^ and the SCF-Cdc4 ubiquitin ligase involved in the stabilization of the replication fork on DNA lesions^81^. Further, as expected from our earlier targeted analysis, we detected changes in assembly for the Lsm1-7–Pat1 complex involved in P-body formation. In addition to these known or expected effects, we observed reorganization of the protein quality control machinery, the endoplasmic reticulum-associated degradation (ERAD) luminal surveillance complex and the 26S proteasome, the nuclear pore complex, both the large and small ribosomal subunits as well as nucleolar complexes, RNA polymerases and the translation regulation machinery. The changes in the protein quality control machinery, particularly in heat-shock factors and the ERAD pathway, suggest that HU treatment challenges proteostasis.

Overall, our data show that combining the FLiP marker library with LiP–MS experiments and network-based analysis globally identifies perturbation-induced changes in protein complexes across the proteome.

### PPI dynamics are linked to Gcn5 acetyltransferase activity

Having established a global picture of protein complex reorganization on DNA replication stress, we observed that 12 of the 56 protein complexes suggested to change their assembly state under these conditions, indicated by a change in at least a single FLiP marker, included known acetylation targets of Gcn5 (ref. ^82^). Gcn5 is the acetyltransferase of the SAGA complex, a protein-modifying complex with deubiquitinase and acetyltransferase activity that is recruited to DNA specifically under stress, leading to gene-specific histone acetylation and transcriptional activation^77,83,84^. It also targets nonhistone proteins, such as NPCs^85^, the INO80 and RSC complexes, RNA polymerases and other complexes that changed assembly state based on our screen, as well as itself^82^. We therefore further investigated the behavior and role of the SAGA complex in HU-treated cells.

Based on a FLiP marker change of its Chd1 subunit, our data suggested reorganization of SAGA itself on HU stress. Since this marker is of low confidence, we orthogonally investigated the rearrangement of SAGA under HU stress by immuno-precipitating FLAG-tagged Ada3, a core SAGA component, from cells grown with and without HU. We quantified interactors with label-free mass spectrometry using data-dependent acquisition (DDA) and tested for differential abundance between the two conditions. Of the 235 interactors we identified (SAINT probability of 1), only the SAGA subunit Spt8 significantly decreased on HU stress (*P* = 0.055, Fig. 4b). This observation is of particular interest because loss of Spt8 is known to characterize the transition from the SAGA to the SAGA-like (SLIK) complex and is induced by C-terminal cleavage of Spt7 (ref. ^86^). To investigate whether this cleavage event occurs, we C-terminally GFP-tagged Spt7 and observed an increase of the C-terminal cleavage product and a decrease of full-length protein on HU stress (Fig. 4c). Our data are thus consistent with a SAGA–SLIK transition occurring in yeast under HU stress^87^.

We went on to investigate the potential functional role of SAGA in the response to HU stress and in the observed rearrangements of protein complexes. Since 12 protein complexes with altered assembly contain at least one known Gcn5 acetylation target, we hypothesized that changes in Gcn5-dependent acetylation could be linked to alterations in the assembly state of these complexes on HU treatment. Although our experiment was not designed to detect acetylated peptides, which are of low abundance and require enrichment to achieve high coverage, we asked whether acetylation changes could nevertheless be detected after HU treatment. Indeed, out of the 160 acetylated peptides we could detect, 15 peptides from 14 proteins changed significantly after correction for protein abundance and multiple testing (ANOVA, adjusted *P* < 0.1, Fig. 4d and Supplementary Table 6). Notably, proteins with increased acetylation on HU stress include the N-termini of histones Htb1 and Htb2, both Gcn5 targets (Supplementary Fig. 5c) and part of the nucleosome, which changes assembly state on HU treatment. We also observed increased acetylation of Nsp1, Tef4 and Pre1, which belong to complexes that we identified as undergoing changes in assembly on HU stress (the nuclear pore, elongation factor eEF1 and the 26S proteasome complex, respectively). For Nsp1 and Tef1 where we identify both an acetylation and a FLiP marker change, none of the altered acetylated peptides overlapped with markers indicating that FLiP marker alterations are not due to acetylation changes within marker sequences. Thus, despite low coverage of acetylated peptides, our data confirmed changes in acetylation patterns of known Gcn5 targets Htb1 and Htb2 (refs. ^82,88^), indicating that acetylation by SAGA is altered under these conditions. This suggests that SAGA may contribute to orchestrating the response to replication stress.

**Fig. 5:**
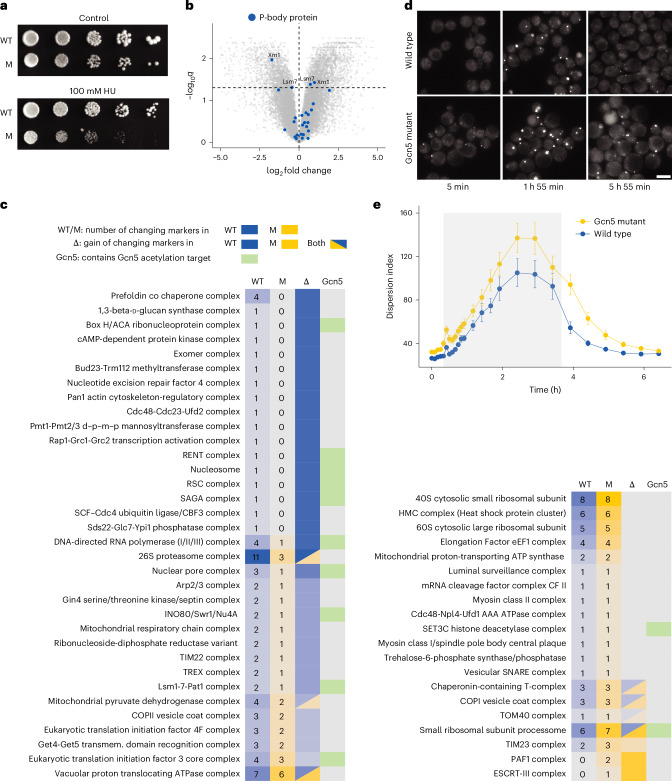
SAGA Gcn5 acetyltransferase activity is linked to protein complex dynamics under DNA replication stress. **a**, Fitness assay of the Gcn5 catalytic dead mutant (M) and wild type (WT) strain grown with 100 mM HU. **b**, Volcano plot showing differential abundance analysis of LiP peptides on HU-induced DNA replication stress in the Gcn5 catalytic dead mutant. Peptide abundances were corrected for protein abundances. *P* values were calculated by an ANOVA (one-way) and corrected for multiple testing (Benjamini–Hochberg). The dotted horizontal line indicates a significance cutoff of 0.05 for adjusted *P* values. Blue dots represent FLiP markers of P-body proteins. **c**, Changes in protein complex network regions identified in the wild type and the Gcn5 catalytic dead mutant on HU stress. The first two columns WT (blue) and M (yellow) indicate the number of changing FLiP markers in the wild type and mutant on HU stress in the respective protein complex. The Δ column is blue if there is a gain in changing FLiP markers in the wild type, yellow if there is a gain in the mutant, gray if there the same markers change in both wild type and mutant and half blue half yellow if different markers change in the wild type and the mutant. The Gcn5 column indicates whether the protein complex network region contains a Gcn5 acetylation target (green) or not (gray)^82^. **d**, Time-lapse fluorescence microscopy comparing P-body formation in wild type and gcn5-E173A cells before (5 min), during (1 h 55 min) and after (5 h 55 min) 200 mM HU stress. Scale bar, 5 μm. **e**, Averaged single-cell quantifications of Dcp2-mNG dispersion index in time-lapse microscopy data during replication stress induced by a pulse of HU (shaded gray area). Error bars indicate 95% confidence intervals (*n* = 440 wild-type cells and *n* = 640 Gcn5 mutant cells were quantified).

To test this idea and probe whether Gcn5-dependent acetylation is needed to regulate the cellular response on DNA replication stress, we used a *gcn5-E173A* mutant strain with a single point mutation that disrupts Gcn5 acetyltransferase activity^89,90^, hereafter termed Gcn5 catalytic dead. The mutant showed reduced viability under HU-induced stress but not under normal growth conditions, indicating that Gcn5 activity is required for an efficient stress response to HU (Fig. 5a).

Next, we harnessed the power of our FLiP library to compare changes in PPIs on HU stress in wild type and Gcn5 catalytic dead cells. We first carried out LiP–MS on the Gcn5 catalytic dead strain in the presence and absence of HU. Out of all 2,406 detected proteins, only 35 changed abundances (ANOVA, |log_2_ fold change| > 0.5, adjusted *P* < 0.1; Supplementary Fig. 6a and Supplementary Table 7). After correcting for protein abundance changes, out of the 20,849 identified LiP peptides reporting on protein structure, 3,705 peptides from 759 proteins significantly changed and thus indicated a protein structural alteration on HU treatment (Fig. 5b, ANOVA, adjusted *P* < 0.05; Supplementary Fig. 6b and Supplementary Table 8). Of these 3,705 LiP peptides indicating structural changes, 1,510 peptides from 352 proteins correspond to FLiP markers and thus report potential changes in PPIs on HU treatment of the Gcn5 catalytic dead strain. In contrast to the wild type yeast, the FLiP-shortlisted hits were not enriched for protein- or RNA-binding domains in this mutant strain (Supplementary Fig. 6c,d).

To compare altered protein complexes between wild type and Gcn5 catalytic dead cells, we only considered FLiP markers detected consistently among both datasets for network propagation, resulting in 54 protein complexes (49 having between one and 12 high confidence marker changes, Supplementary Table 9). We found 13 protein complexes with exactly the same FLiP marker changes in wild type and mutant cells, highlighting the method’s ability to identify a robust subset of protein complexes that are independent of Gcn5 acetyltransferase activity (Fig. 5c and Supplementary Fig. 6e). These complexes include the protein quality control machinery such as the HMC complex (heat-shock protein cluster) and the ERAD pathway (luminal surveillance complex), in line with the notion that the proteostasis defects caused by HU treatment are a direct consequence of incomplete replication and not of Gcn5 activity.

We identified four protein complexes (Small ribosomal subunit processome, TIM23, ESCRT-III, PafI) that gained at least one FLiP marker in Gcn5 catalytic dead cells relative to wild type. These may be associated with alternative pathways activated to cope with DNA replication stress in the absence of Gcn5 activity or rearrangements in protein complexes that are usually inhibited by functional Gcn5.

In contrast, 34 protein complexes lost at least one FLiP marker change in Gcn5 mutant relative to wild type cells, and ten of these complexes contain known Gcn5 acetylation targets, indicating that these complex rearrangements observed on HU stress depend directly or indirectly on the acetyltransferase activity of the SAGA or SLIK complexes. In line with this hypothesis, neither acetylation changes of the known Gcn5 targets Htb1 and Htb2 nor FLiP marker changes of the associated nucleosome were observed in the mutant on HU stress (Supplementary Fig. 6f). Additionally, Gcn5 mutant cells showed no FLiP marker change in the SAGA complex itself, which is also known to acetylate its Ada3 subunit under stress^91^. Although we did not detect the acetylated Ada3 peptide in an automated search, a manual search of our data confirmed that acetylation increases on HU stress in wild type cells but is not identified in the Gcn5 catalytic dead mutant (Supplementary Fig. 6g,h), highlighting another example where both acetylation of a known Gcn5 target and the associated protein complex rearrangement are lost in the mutant. Together, our data suggest we have identified protein complexes that rearrange under DNA replication stress in a manner that is directly or indirectly dependent on Gcn5 activity.

The Lsm1-7–Pat1 complex, linked to P-body formation, lost FLiP marker changes in Gcn5 catalytic dead cells. For nine out of the 13 most abundant P-body proteins detected in both datasets, six had changing FLiP markers in the wild type, compared to only two proteins in the mutant (Fig. 5b). This suggests that P-body formation may be dysregulated in the Gcn5 mutant and that there is a link between P-body formation and SAGA acetyltransferase activity. To test this, we monitored the dynamics of P-body formation on HU stress with time-lapse fluorescence microscopy of mNeonGreen-tagged (mNG) Dcp2 strains in wild type and Gcn5 catalytic dead backgrounds^92^. Even in the absence of HU stress, we observed small P-bodies in Gcn5 catalytic dead cells, but not in wild type cells (Fig. 5d). Further, quantification of Dcp2-mNG clustering on HU treatment showed that P-bodies formed with similar dynamics in both wild type and mutant cells (Fig. 5e). However, in mutant cells, more and/or larger P-bodies formed, and these P-bodies disassembled more slowly than in wild type cells when HU was removed (Fig. 5d,e). FLiP–MS possibly detects fewer PPI changes among P-body components in HU-treated Gcn5 catalytic dead cells because P-bodies are already present to some degree before replication stress. Thus, our data suggest that the acetyltransferase activity of Gcn5 directly or indirectly inhibits P-body formation.

Overall, these results demonstrate that our approach is scalable and can be used to identify changes in protein complex assembly under different conditions and across different genetic backgrounds, all in a single experiment. Our data show that Gcn5-dependent acetylation directly or indirectly affected assembly of ten protein complexes with known Gcn5 acetylation targets. They also uncover a link between SAGA acetylation activity and the formation of P-bodies.

## Discussion

The FLiP–MS approach combines systems and structural analysis of protein complexes, by enabling global identification of changes in PPIs under a perturbation of interest while simultaneously providing structural information about the involved PBIs. We have demonstrated the power of this high throughput, proteome-wide and structurally informative approach in a global and high-resolution analysis of interactome changes in yeast responding to DNA replication stress. We identified expected changes in the assembly state of P-bodies and of other protein complexes involved in remodeling chromatin and reorganizing RNA turnover, providing strong support for the ability of FLiP–MS to identify genuine biological events. Further, we provide a global view of the role of the SAGA complex in orchestrating the cellular response to replication stress, including an unexpected connection with P-body formation.

FLiP–MS is a modular approach, with potential applications across various research fields. The first step generates a proteome-wide library of marker peptides that report on PPI changes in native cell extracts, which we termed the FLiP marker library. We have shown that the library is enriched for known PBIs and also includes new PBIs that are so far not represented by experimental structures. In addition, the library can include markers of structural rearrangements or posttranslational modifications at regions other than the PBI, which accompany the formation or dissociation of a protein complex. The FLiP library can be used in different structural biology applications as well as in fields where the lack of high-resolution protein complex structures is limiting. For instance, the library could be used to train or evaluate multimeric protein structure predictions or to design mutations within PBIs, or compounds targeting them, for functional characterization of protein complexes^93,94^. FLiP marker libraries could be generated for different species, including human, to help understand the effect of mutations associated with Mendelian diseases and cancer, which are known to be enriched in PBIs^3,4,11^, and to support drug development. We have annotated each marker according to current evidence for location at a PBI, which can be considered in downstream applications of this resource.

The FLiP library complements previously available structural data as it is enriched in disordered regions, which are typically underrepresented in X-ray crystal structures^95^. Over 70% of yeast proteins are moderately or highly disordered^96^, and disordered regions are known to form interaction interfaces^46^ and are enriched in interaction-rich hubs in PPI networks^97–99^. Thus, the FLiP marker library should improve the study and structural prediction of protein complexes involving disordered regions, for which training data are sparse. Last, the library also covers protein–RNA interaction interfaces, which could be valuable information for RNA biology and help in drug development for targeting protein–RNA interactions^100^.

The second step of our approach makes use of the FLiP marker library to track PPI changes under conditions of interest from unfractionated cell lysates by integrating it with an independent LiP–MS analysis. This can be valuable in generating hypotheses on how specific PPIs may be involved in the response to a particular perturbation. In this type of application, both markers at PBIs and those distant from them can help identify proteins changing their assembly state, and the potential PBI location information in particular can be leveraged to test these hypotheses via mutation or designed drugs. In our own work, we integrated our FLiP library comprising 6,441 markers of 1,086 proteins (covering one-sixth of the yeast proteome) with LiP–MS analysis of yeast cells under HU-induced DNA replication stress and captured interaction changes for 566 proteins in situ. The number of hits suggests that PPI changes are a central aspect of the cellular response.

We showed that our data captured changes in the assembly state of components of P-bodies. For the proteins Pat1, Dhh1, Dcp2, Xrn1 and the Lsm complex, the detected interaction changes match known functional and structural data, thus validating the ability of our method to capture changes in protein complexes.

Our FLiP–MS data suggest that the SAGA complex itself undergoes reorganization under replication stress, based on a change in a single low confidence FLiP marker mapping to the SAGA component Chd1. Follow-up experiments further indicate that HU stress induces a transition from the SAGA to the SLIK complex, involving a loss of the Spt8 subunit and cleavage of Spt7. The SAGA and SLIK complex components are too large to be detected by our FLiP pipeline, which is likely why we do not see marker changes for subunits involved in this transition. Chd1 is separated in a fraction of lower-than-expected molecular weight, possibly reflecting cleavage events. The FLiP marker of Chd1 maps to its DNA binding domain and may therefore represent a protein–DNA interaction marker. While intriguing, given the known function of Chd1 to recruit SAGA or SLIK to DNA^87^, understanding the relevance of this marker for SAGA assembly will require further experiments.

In the third step of our approach, the list of affected proteins is projected on a PPI network thus suggesting protein complexes that change their assembly state. This increases the biological interpretability of the results and supports the generation of mechanistic hypotheses on complexes that change their assembly state on perturbation. Our data indicate rearrangements of 56 protein complexes on DNA replication stress, and shows how, given the interconnected and small-world nature of PPI networks, even a set of FLiP markers mapping to a relatively small number of proteins (roughly 1,000) can capture changes throughout the network. In general, changing FLiP markers should be the starting point for hypotheses about altered protein complex dynamics that must subsequently be orthogonally validated. The use of network analysis will help prioritize complexes with multiple lines of evidence for validation, thus reducing the impact of false positives.

We detected reorganization of several protein complexes known to function in protein quality control, suggesting HU-induced proteostasis effects. This could in turn explain the response we observed in the translation machinery and ribosome biosynthesis, which are among the first processes to be inhibited in response to global stresses. We rationalize the activation of protein quality control in response to HU treatment as the consequence of unequal DNA replication along the genome leading to a pseudo-aneuploid state of the cell, where some genomic regions are replicated and others not, causing an imbalance in copy numbers for components of protein complexes, as in standard aneuploidy^101^. Indeed, aneuploidy is a major source of proteostasis defects and proteotoxic stress^102^ and leads to robust engagement of the protein folding machinery, which is required for survival^101,103^. In support of our interpretation that partial DNA replication affects proteostasis, mutations affecting the folding machinery render cells more sensitive to HU^104,105^.

Our data also allowed us to hypothesize an effect of the SAGA acetyltransferase Gcn5 on multiple PPI changes since 12 out of the 56 complexes include at least one Gcn5 acetylation target. By investigating Gcn5 mutant cells, we observed both Gcn5-dependent changes in acetylation patterns of known (Htb1, Htb2 and Ada3) and, to our knowledge, previously unreported (Nsp1 and Pre1) Gcn5 targets as well as Gcn5-dependent changes in the assembly state of complexes involving these proteins (nucleosome, SAGA, NPC and 26S proteasome). This suggests a direct or indirect regulation of assembly state of these complexes by Gcn5. Further, we identified an additional eight protein complexes with known Gcn5 acetylation targets that changed assembly state on HU stress in a Gcn5-dependent manner. These include chromatin remodeling complexes, as well as several complexes involved in ribosome biogenesis and translation, suggesting that both the stabilization of the replication fork in response to replication stress and the inhibition of ribosome synthesis, that we suspect are a general response to proteostatic stress, might be under the direct control of SAGA activity. Our results also indicate that Gcn5 affected PPI changes related to mRNA processing events and connect the SAGA complex to P-body formation.

Other clusters with Gcn5-dependent PPI changes induced by HU treatment are not known to include SAGA targets, and many of them (that is, the prefoldin cochaperone complex, the SCF-Cdc4 ubiquitin ligase, several components of the secretory pathway, the proteasome and the mitochondrial protein import machinery) consist of cytoplasmic or organellar membrane proteins and are therefore unlikely to be targets of nuclear-localized SAGA. However, Gcn5 itself was proposed to be present both in the cytosol^106^ and in mitochondria^107^, which may explain the observed changes, or they might result from secondary or downstream effects. These findings show how FLiP–MS enables hypothesis generation by providing a graspable birds-eye view of interactome changes.

The FLiP–MS workflow has advantages over existing methods to probe PPIs, notably that it scalably provides structural information about altered protein complexes^42^. Crosslinking coupled to mass spectrometry also provides structural information and has the important advantage that it directly probes protein interaction sites and identifies interaction partners. XL–MS is complementary to FLiP–MS in this regard. However, when applied in complex samples, it suffers from low sensitivity^108^ and an even stronger bias than other MS-based approaches toward highly abundant proteins due to the low abundance of interprotein crosslinks^19^. This can be mitigated with the use of fractionation techniques, but at the expense of scalability. For instance, Liu et al. found 326 PPIs in HeLa lysates by strong cation exchange fractionation and acquiring 20 fractions per sample by MS with a 3-h gradient^109^. In this experiment, the number of probed PBIs (413 interprotein crosslinks for 326 PPIs, 5% false discovery rate (FDR)) detected by XL–MS is considerably lower than in the FLiP–MS workflow (6,441 FLiP markers for 1,068 proteins, 5% FDR) and the number of runs per sample is 20 times higher compared to the FLiP approach. Recent advances boosting sensitivity and increasing throughput of XL–MS via enrichable crosslinkers that can be used in vivo may enable direct study of in situ PPI dynamics in the future^110,111^. Hydrogen-deuterium exchange–MS is powerful for the analysis of protein dynamics but is currently applicable only to purified proteins^112^. CF–MS and AP–MS are established methods to study PPIs and provide valuable information about involved interaction partners, but they do not contain structural information and suffer from poor scalability. For instance, in their seminal study, Huttlin et al.^15^ analyzed more than 15,000 AP–MS experiments comparing two cell lines, whereas comparing two cellular states using our FLiP–MS library only requires 16 MS runs. This combination of structural information and scalability permits the study of many cellular responses at reasonable throughput and the generation of hypotheses on protein complexes altered under the studied conditions, thus helping to understand the consequences of PPI changes.

Nevertheless, our workflow has shortcomings. First, protein size and the strength of PPIs determine the set of proteins that can be separated by serial ultrafiltration. Proteins for which the sizes of monomeric and complex-bound forms cannot be separated with the given MWCOs will localize in the same fraction. Similarly, the workflow may also fail to cover stable or unstable interactions if they either do not dissociate or fully dissociate during the ultrafiltration step and interpretation of low confidence FLiP markers could be complicated in general because of cleavage events or other features that cause a protein to fractionate in an unexpected way. In addition, the method requires MS-based detection of relevant protein regions both for library generation as well as for the detection of library peptides in LiP–MS experiments. Thus, it is biased toward abundant proteins, similar to standard MS-based proteomics. Finally, the use of previous knowledge might bias the outcome toward more studied proteins, a frequent problem in systems biology analyses.

We envision that FLiP–MS profiling across multiple conditions will be used side-by-side with genomic, transcriptomic and classical proteomic measurements, and that the resulting complementary information will substantially increase our capability to generate biological hypotheses and our understanding of cellular events. As more protein complexes are structurally characterized, the more markers in any FLiP–MS library will be independently validated and putative interaction partners identified. To increase coverage, the FLiP–MS library can be extended to other species, and stress-specific libraries would allow interactions that emerge during stress to be captured. Such extensions will move us toward comprehensive measurement of the state of a cell and thus enable system-wide understanding.

## Methods

### Cultivation of yeast cells

#### FLiP library

A 50-ml preculture of *Saccharomyces cerevisiae* (strain BY4716) was grown in a 250-ml Erlenmeyer flask from a single colony in yeast extract peptone dextrose at 30 °C with constant 150-rpm shaking for 6 h. The preculture was diluted 1:200 in 4 l of yeast extract peptone dextrose and equally distributed onto four 5-l Erlenmeyer flasks. The cultures were grown to an optical density at 600 nm of 0.8 ± 0.1.

#### HU stress

Four colonies of *Saccharomyces cerevisiae* (wild type BY4741 *his3Δ1 ura3Δ0 leu2Δ0, met15Δ0*; Gcn5 mutant BY4741 *gcn5-E173A his3Δ200 ura3-52 leu2Δ0 lys2-801 ade2-101 trp1Δ63*; Ada3-3xFLAG BY4741 MATa *his3Δ1 leu2Δ0 ura3Δ0 met15Δ0 ada3-3xFLAG:kanMX6*; Spt7-GFP BY4741 MATa *his3∆1 leu2Δ0 ura3Δ0 met15Δ0 spt7-GFP:HIS3* from the yeast GFP collection^113^) were grown to an optical density at 600 nm of 0.8 ± 0.1 in synthetic complete 2% glucose (SC + glucose, CSM complete 40 ADE, Formedium DCS0039, Yest nitrogen base without amino acids, Formedium CYN0410, d-(+)-glucose, Sigma-Aldrich, cat. no. G7021) at 30 °C with constant 160-rpm shaking. For HU stress, at an optical density at 600 nm of 0.8 ± 0.1 the cells were transferred into 50 ml Falcon tubes (CELLSTAR, cat. no. 227261), spun down (1,000*g*, 5 min, 4 °C) and resuspended, washed once and resuspended in SC + 2% glucose medium supplemented with 200 mM HU (Sigma-Aldrich, cat. no. H8627). The cultures were incubated for 2 h at 30 °C with constant 160-rpm shaking.

### Harvesting of yeast

#### FLiP library generation

The cultures were equally distributed onto eight 500-ml Corning polypropylene centrifuge tubes and spun down at 3,428*g* for 15 min at room temperature. Every batch was washed twice with 100 ml of PBS and spun down with the same parameters. Each pellet was dissolved in 5 ml of LiP buffer (20 mM HEPES, Sigma H4034, 150 mM KCl, Merck 1049360250 and 10 mM MgCl_2_, Sigma M2670 at pH 7.5, all components from Sigma-Aldrich) at room temperature and equally distributed into six 1.5 ml Eppendorf tubes. The cells were pelleted by centrifuging at 800*g* for 5 min at room temperature. The supernatant was discarded, and the cells were snap frozen in liquid nitrogen and stored at −80 °C.

#### HU stress

Here, 25 ml of each culture were collected in Falcon tubes (CELLSTAR, cat. no. 227261) and collected by centrifuging at 1,000*g* for 5 min at 4 °C. The pellets were washed with 10 ml PBS and centrifuged with the same parameters. The pellets were then resuspended in 1 ml of LiP buffer supplemented with 1× Roche Complete Protease Inhibitor EDTA-free (Sigma-Aldrich, cat. no. 11873580001) and transferred into 1.5-ml Eppendorf tubes. The cells were pelleted by centrifuging for 5 min at 800*g* at room temperature. The supernatant was discarded, and the cells snap frozen in liquid nitrogen and stored on ice before for processing.

### Cell lysis

The yeast pellets were resuspended in LiP buffer with 1× Roche Complete Protease Inhibitor EDTA-free (Sigma-Aldrich, cat. no. 11873580001) and transferred into screwcap microcentrifuge tubes. A volume of glass beads equal to the volume of resuspended cells was added to each tube. The cells were lysed at 4 °C in a benchtop homogenizer (MP Biomedicals, Fastprep-24 5G) by eight cycles of 30-s beating at 5.5 Hz followed by a 200-s break. The microcentrifuge tubes were perforated with a hot needle on the bottom and the top of the tube. The tubes were positioned onto 1.5-ml Eppendorf tubes and spun at 800*g* for 30 s. The lysates were cleared of cell debris by centrifuging for 15 min at 21,500*g* at 4 °C. The supernatant was collected.

### Serial ultrafiltration and RNase treatment

During serial ultrafiltration, we consecutively filtered the lysate through ultrafiltration devices with decreasing MWCOs. The retentates were collected, and the filtrates transferred onto the next highest MWCO filter. The process was repeated until the lowest MWCO filter was reached. Here we used 50-ml Amicon Ultra filters with MWCOs of 100, 50, 30 and 10 kDa (Sigma-Aldrich, cat. nos. UFC910008, UFC905008, UFC903008, UFC901008). All filters were washed with 5 ml of LiP buffer by centrifuging at 1,500*g* for 5 min immediately before loading the filters with lysate. All centrifugation steps were conducted at 4 °C. For good size separation, Amicon filters must be used sequentially, rather than using one or two consecutive cutoffs^114,115^.

Given the importance of RNA-binding proteins in the organization of cellular protein networks, we designed our FLiP library to capture changes in RNA-dependent protein–protein and protein–RNA-binding events, in addition to PPIs in general. We did this by diluting the lysate with LiP buffer with 1× Roche Complete Protease Inhibitor EDTA-free to 40 ml and adding 143 μl of RNase A (10 mg ml^−1^ stock solution in 10 mM sodium acetate, pH 7.4, Merck, R4875), RNase H (5,000 U ml^−1^, NEB, M0297), RNase I (10 U µl^−1^, Thermo Fisher Scientific, EN0601), RNase III (1 U µl^−1^, Thermo Fisher Scientific, AM2290) and RNase T1 (1,000 U µl^−1^, Thermo Fisher Scientific, EN0542). Samples were incubated at 4 °C on an analog tube roller for 1 h. These conditions were previously used to identify protein–RNA complexes confidently and at a proteome-wide scale by shifts in density gradient ultracentrifugation profiles between control and RNase treated samples^116^. Our rationale was that digestion of RNA will result in destabilization of protein–RNA complexes and RNA-dependent protein–protein complexes that can then be separated by serial ultrafiltration and will thus contribute FLiP markers. This requires partial digestion of RNA such that some of the protein–RNA complex remains intact and is retained in a high molecular weight fraction, whereas some of it disassembles and is in a lower molecular weight fraction. We assessed whether this is the case for ribosomal proteins and found that for 16 proteins of the 60S large ribosomal subunit (RPL10, RPL13A, RPL13B, RPL16B, RPL22A, RPL25, RPL28, RPL36B, RPL4A, RPL5, RPL6A, RPL6B, RPL7A, RPL7B, RPL8A, RPL8B) and for 18 proteins of the 40S small ribosomal subunit (RPS0B, RPS12, RPS14A, RPS15, RPS1A, RPS1B, RPS2, RPS20, RPS21A, RPS21B, RPS26A, RPS28A, RPS29A, RPS3, RPS31, RPS5, RPS7A, RPS7B), there was at least one FLiP marker between the 100-K and a lower molecular weight fraction. This suggests that the conditions we used enable the identification of FLiP markers of protein–RNA complexes or RNA-dependent protein–protein complexes.

After RNase treatment, four 50-ml Amicon Ultra filters with a MWCO of 100 kDa were loaded with 10 ml lysate, resulting in four replicates. Besides size fractionation, our goal during centrifugation was to concentrate the retained proteins while avoiding precipitation. For the different MWCO filters of 100, 50, 30 and 10 kDa. Retentate volumes of 1.5–2 ml, 200–300 μl, 200–300 μl and 100–300 μl, respectively, were achieved by centrifuging at 1,500*g* for 1 h, 30 min, 30 min and 1.5 h, respectively. For the 100 K fraction, we needed to ensure that the retentate did not become overconcentrated, which would lead to aggregation. To prevent aggregation, the volume was reduced to ~2 ml, requiring ~1 h of centrifugation. This is comparatively long because this fraction has a high initial protein concentration, which makes the filtration process slow. For the 50-K and 30-K fractions, the filtration process is much faster and a retention volume of ~200 μl can be reached within 30 min. For the 10-K fraction, the filtration process is again comparatively slow due to the small pore size. However, especially for this fraction, it is extremely important to concentrate the samples, since this is the fraction with the lowest total protein amount. Thus, we filtered for the 10-K fraction for 1 h 30 min to reach maximal concentration.

We added 83, 18, 3 and 3 μl of 50× Roche Complete Protease Inhibitor EDTA-free (Sigma-Aldrich, cat. no. 11873580001), respectively, to the retentates of the decreasing MWCO fractions to inhibit protease activity. We stored the retentates on ice in a 4 °C until further processing.

### Protein concentration measurement

Protein concentrations of the lysate, as well as the fractions, were quantified with a bicinchoninic acid assay (Pierce BCA Protein Assay Kit, Thermo Fisher Scientific, ref. 23225).

#### SEC of different filter fractions

A single replicate of yeast lysate was fractionated by serial ultrafiltration as described above. Each fraction was filtered through a 2-ml Spin-X centrifuge tube filter (0.22 μm cellulose acetate) for 30 s with 800*g* at 4 °C. Concentration of each fraction was assessed by BCA and 100 μg of native proteins were injected to a SEC-s4000 column (300 × 7.8 mm, pore size 500 Å, particle size 5 μm, BioSep). Proteins were separated by SEC with LiP buffer at a flow rate of 500 μl min^−1^ for 45 min at 4 °C. Fraction collection was performed from 9 min to 33 min at 0.25 min per fraction. Only ultraviolet traces of eluting proteins were analyzed and are shown here, collected fractions were not further processed.

### Native PAGE of protein assemblies in different filter fractions

A single replicate of yeast lysate was fractionated by serial ultrafiltration as described above and 100 μg of native proteins from each fraction were separated by size two different NuPAGE gels (NuPAGE Tris-Acetate Mini Protein Gels, 3 to 8%, Thermo EA0376BOX, NuPAGE Tris-Acetate Mini Protein Gels, 7%, Thermo EA0355BOX). Briefly, 7.5 μl sample was mixed with 2.5 μl 4× native PAGE sample buffer and separated on a NativePAGE gel (Thermo, BN2003) using the dark blue cathode buffer and applying 150 V for 60 min followed by 250 V for 30 min at 4 °C. Coomassie from the cathode buffer was fixed by microwaving the gel in fixing solution (40% methanol, Fisher Scientific 15631400, 10% acetic acid, Sigma-Aldrich 45754) for 45 s at ~1,000 W. The gel was shaken for 15 min at room temperature. For destaining, the gel was transferred into 100 ml 8% acetic acid solution and microwaved for 45 s at ~1,000 W and was kept in the destaining solution overnight. Gels were imaged on a Vilber Fusion FX with the Fusion FX6 Edge software using the ‘Coomassie optimal’ settings.

### Sample randomization and blinding

The samples were randomized after adjusting concentration. The randomized order was kept for the rest of the protocol including MS data acquisition. The randomized samples were only labeled with numbers. Different investigators performed randomization and limited proteolysis as well as the tryptic digest to ensure blinding.

### Limited proteolysis

The optimal protein concentration for LiP–MS is 2 mg ml^−1^ (ref. ^30^). However, to compare between filter fractions, we needed to adjust the protein concentrations of all samples to 1.3 mg ml^−1^, which is the lowest concentration across all filter fractions (found in the 10-K fraction). Limited proteolysis was performed as described previously^31^. Briefly, 50 μl of each sample were transferred into a PCR tube and heated to 25 °C for 5 min in a thermocycler (Biometra TRIO 48, 2070723). PK (Tritirachium album, 10 mg, Sigma-Aldrich, P2308) was added at an enzyme-to-substrate ratio of 1:100 (w/w) and incubated at 25 °C for 5 min. The digestion was stopped by heating the samples for 5 min at 99 °C. Tryptic control samples were generated in parallel except that 5 μl of water was added instead of PK. Samples were cooled on ice for 5 min. Proteins were denatured by adding a 10% (wt/vol) stock of sodium deoxycholate (DOC, Sigma 30970) to a final concentration of 5% (wt/vol) in a 2-ml 96-well plate.

### Tryptic digest

Disulfide bonds were reduced by adding a stock of 200 mM tris-(2-carboxyethyl)-phosphine (TCEP, Sigma C4706) to a final concentration of 5 mM. The reaction was incubated at 37 °C for 40 min with 200-rpm shaking. The reduced disulfide bonds were alkylated by adding a freshly prepared stock of 1 M iodoacetamide (Sigma I1149) to a final concentration of 40 mM. The reaction was incubated at room temperature for 25 min without shaking and protected from light. The samples were diluted 1:5 with a 100 mM ammonium bicarbonate solution (Sigma-Aldrich A6141) to yield a DOC concentration of 1% (wt/vol). Finally, 1 μl of lysyl endopeptidase R (FUJIFILM Wako Pure Chemical Corporation, 129-02541) and 2 μl of trypsin (0.5 mg ml^−1^, Promega, V511C) were added. The samples were incubated at 37 °C overnight with 200-rpm shaking. The reaction was stopped the following day by adding 100% formic acid (Carl Roth GmbH) to a concentration of 3% (vol/vol) resulting in a pH below 2. DOC was removed by filtering the samples through a FiltrEX 96-well 0.2 μm polyvinyl difluoride (PVDF) membrane white filter plate (Corning, ref. 3508) by centrifuging at 2,000*g* for 2 min.

### C18-cleanup and sample preparation

A 96-well C18 spin column plate (Nest Group, S8VL) was connected to a vacuum pump and was washed with 200 μl methanol (Fisher Scientific 15631400), followed by 100 μl buffer B (50% acetonitrile (ACN), Fisher Scientific A955-212, 0.1% formic acid, Carl Roth GmbH) and 3× 200 μl buffer A (5% ACN, Fisher Scientific A955-212, 0.1% formic acid, Carl Roth GmbH). Samples were loaded and washed with 3× 200 μl buffer A. Peptides were eluted with 3× 100 μl buffer B and dried in a vacuum centrifuge. All samples were resuspended in buffer A to a peptide concentration of 2 mg ml^−1^. iRT peptides (10×, Biognosys, Pp-2005) were added to the samples in a 1:30 dilution. For each combination of condition and/or fraction and sample type (LiP, tryptic control), a library sample consisting of an equal amount of peptide from every replicate was prepared.

### Affinity-purification mass spectrometry

Yeast expressing ADA3-3xFLAG (BY4741 MATa *his3Δ1 leu2Δ0 ura3Δ0 met15Δ0 ada3-3xFLAG:kanMX6*) and wild-type control yeast cells (BY4741) were grown and gathered each in quadruplicates under control and HU stress as described above. Cell lysates were supplemented with 2.5 μl of benzonase (Sigma-Aldrich, E1014-5KU) and incubated under end over end rotation for 30 min at 4 °C. The postnuclear fraction was isolated by centrifugation at 21,000*g*, 4 °C, 30 min and the supernatant transferred to a new precooled Eppendorf tube. Protein concentrations were determined by Bradford assay and lysates were subsequently adjusted to a protein concentration of 2 mg ml^−1^ in lysis buffer. 0.8–1 mg lysate was used for affinity purification. To this end, samples were precleared with 30 μl of a 50% mouse IgG-agarose bead slurry (A0919, Sigma-Aldrich) for 60 min under end over end rotation at 4 °C. Beads were removed by centrifugation at 1,000*g* for 5 min. ADA3-3xFLAG was affinity purified from the precleared lysate by incubation with 25 μl of a 50% anti-FLAG-M2 bead slurry (A2220, Millipore) for 3 h under end to end rotation at 4 °C. ADA3-3xFLAG-depleted lysate was removed by centrifugation at 1,000*g*, 4 °C for 2 min and beads were washed twice with lysis buffer (without Roche protease inhibitor), followed by three washes with wash buffer (25 mM HEPES-KOH, Sigma H4034, pH 7.4, 100 mM KCl, Merck 1049360250, 2 mM MgCl_2_, Sigma M2670). Each washing step was performed by end over end rotation for 5 min at 4 °C, followed by centrifugation at 1,000*g*, 4 °C for 2 min. Purified material was eluted in 60 μl of 8 M urea (Sigma-Aldrich U5378), 100 mM ammonium bicarbonate (Sigma-Aldrich A6141) by shaking at 1,500 rpm and 37 °C on a thermomixer (Eppendorf) before centrifugation (1,000*g*, 25 °C, 2 min) and transfer of the supernatant to ProteinLoBind tubes. Samples were prepared for mass spectrometry by reduction with TCEP (Sigma C4706) and iodoacetamide-mediated alkylation (Sigma I1149), followed by dilution to 1 M urea with 100 mM ammonium bicarbonate (Sigma-Aldrich A6141). Each sample received 1 μg of trypsin and 1 μg of Lys-C and the digest was allowed to proceed at 37 °C for 20 h before acidification to pH 2 with formic acid. Samples were centrifuged at 21,000*g* for 20 min at 25 °C and desalted using Stage Tips (330 μg binding capacity) before resuspension in 15 μl of buffer A + iRT peptides (1:30).

### LC–MS instrumentation

Samples were analyzed on an Orbitrap Fusion Lumos Tribrid mass spectrometer (Thermo Fisher) equipped with a nano-electrospray ion source and Waters Acquity M-Class UPLC system. Peptides were separated on a 40 cm × 0.75 μm i.d. column (New Objective, PF360-75-10-N-5) packed in house with 1.9-μm C18 beads (Dr. Maisch Reprosil-Pur 120). Liquid chromatography (LC) fractionation was achieved with the following gradients of buffer A (0.1% formic acid, Sigma-Aldrich 33015) and buffer B (99% ACN, Fisher Scientific A955-212, 0.1% formic acid, Sigma-Aldrich 33015): a linear gradient from 5 to 35% buffer B over 120 min, followed by 5 min with an isocratic constant concentration of 90% buffer B. The flow rate was 300 nl min^−1^, and the column was heated to 50 °C.

AP–MS samples were analyzed on an Orbitrap Eclipse Tribrid mass spectrometer (Thermo Fisher Scientific) equipped with a nano-electrospray ion source and a nano-flow LC system (Easy-nLC 1200, Thermo Fisher Scientific). Peptides were separated on a 40 cm × 0.75 mm i.d. column (New Objective, PF360-75-10-N-5) packed in house with 1.9-μm C18 beads (Dr. Maisch Reprosil-Pur 120). LC fractionation was achieved with the following gradients of buffer A (5% ACN, Fisher Scientific A955-212, 0.1% formic acid, Sigma-Aldrich 33015) and buffer B (95% ACN, Fisher Scientific A955-212, 0.1% formic acid, Carl Roth GmbH): a linear gradient from 3 to 30% buffer B over 120 min, followed by 5 min with an isocratic constant concentration of 90% buffer B. The flow rate was 300 nl min^−1^, and the column was heated to 50 °C.

### DDA

For shotgun LC coupled with tandem MS (LC–MS/MS) DDA on the Orbitrap Fusion Lumos Tribrid mass spectrometer, 2 μl from each library sample were injected. MS1 spectra were acquired from 350 to 1,500 *m*/*z* at an orbitrap with a resolution of 120,000 with an automated gain control (AGC) target of 150% or 100-ms injection time. Precursors with an intensity exceeding 50,000 and a positively charged state between 2 and 5 were selected for data-dependent MS2 scans. Dynamic exclusion was applied after a single occurrence for 30 s with a 10-ppm mass tolerance. Selected precursors were isolated with a quadrupole and an isolation window of 1.2 *m*/*z*. Precursors were fragmented with high-energy collision-induced dissociation (HCD) with a fixed collision energy of 27%. MS2 spectra were acquired at an orbitrap resolution of 30,000, an automatically adapting scan range (minimum − precursor × charge + 10.0), and an AGC target of 200% or a dynamic injection time that automatically calculates the maximal time available. All MS data were collected as raw files using Xcalibur (v.4.2).

For shotgun LC–MS/MS DDA of the AP–MS data on the Orbitrap Eclipse Tribrid mass spectrometer, 4 μl from each sample were injected. MS1 spectra were acquired from 350 to 1,400 *m*/*z* at an orbitrap with a resolution of 120,000 with an AGC target of 200% or 54-ms injection time. Precursors with an intensity exceeding 50,000 and a positively charged state between 2 and 7 were selected for data-dependent MS2 scans. Dynamic exclusion was applied after a single occurrence for 20 s with a 10-ppm mass tolerance. Selected precursors were isolated with a quadrupole and an isolation window of 0.7 *m*/*z*. Precursors were fragmented with HCD with a fixed collision energy of 30%. MS2 spectra were acquired at an orbitrap resolution of 30,000, an automatically adapting scan range (minimum − precursor × charge + 10.0), and an AGC target of 200% or a maximum injection time of 54 ms. All MS data were collected as raw files using Xcalibur (v.4.3).

### DIA

Aliquots of 2 μl of each sample were injected independently and measured in DIA mode. The DIA-MS method consisted of a survey MS1 scan from 350 to 2,000 *m*/*z* at a resolution of 120,000 with an AGC target of 50% or 100-ms injection time, followed by DIA in 41 variable-width isolation windows. The *m*/*z* isolation ranges are listed in Supplementary Table 10. Precursors were isolated by a quadrupole and fragmented with HCD with a collision energy of 28%. DIA-MS2 spectra were acquired with a scan range of 200 to 1,800 *m*/*z* at an orbitrap resolution of 30,000 with an AGC target of 200% or 54-ms injection time.

### Data analysis

The code for the described data analysis pipelines is publicly available on GitHub^117^.

### Search engines

The data of the serial ultrafiltration experiment generating the FLiP marker library was searched in Spectronaut v.15.6.211220.50606 and for all other experiments in Spectronaut v.17.1.221229.55965 (Biognosys). Hybrid libraries for the tryptic control and the LiP samples consisting of the corresponding DDA and DIA runs were created based on a Pulsar search using the default settings, with the exception of digest type, which was set to ‘semi-specific’ for the LiP samples only, and the minimal peptide length, which was set to six. The data were searched against the UniProt fasta database (strain S288c, UP000002311, July 2019). For the serial ultrafiltration experiment, the targeted data extraction was performed in Spectronaut 15 with default settings (BGS Factory Settings) except for the data filtering, which was set to ‘Qvalue percentile’ with a cutoff of 0.75. For all other experiments analyzed in Spectronaut 17, default settings were used. In all experiments, the FDR was set to 1% on precursor and protein levels and no values were imputed. The LiP and tryptic control samples were searched separately. From the LiP search, we exported peptide intensities, and from the tryptic control search, we exported protein intensities.

For the acetylation analysis, hybrid libraries for the tryptic control samples were generated with the same settings as above with an additional variable modification ‘Acetyl (K)’. The data was extracted using Spectronaut v.17.1.221229.55965 with default settings and imputation set to ‘Use Background Signal’. This ensures to include acetylated peptides that are present in only one condition.

The AP–MS data were searched in Spectromine v.3.2.22022.52329 (Biognosys). The search was performed with default parameters except for the proteotypicity filter, which was set to ‘Proteotypic only’ and normalization, which was not applied. The data were searched against the UniProt fasta database (strain S288c, UP000002311, July 2019) and a list of typical contaminants. The FDR was set to 1% on peptide and protein levels. For SAINT analysis, the control and HU-stressed samples were searched separately but together with their respective wild-type backgrounds and the quantification of proteins was exported based on number of spectral matches. For differential abundance analysis, control and HU-stressed samples and their respective backgrounds were searched together, and the quantification of proteins was exported as intensities.

### Manual peak extraction

The acetylated peptides of Ada3 were manually extracted from the DIA files of the tryptic control using Skyline 64-bit (v.21.1). The MS/MS filtering was adjusted to the DIA method described above. All possible b- and y-ions up to a precursor charge minus one were extracted and manually filtered for coelution.

### Data filtering

For all experiments, we required a peptide in the LiP dataset and its corresponding protein in the tryptic control dataset to be measured in at least biological triplicates in at least two conditions and/or fractions to be considered for further downstream analysis.

### Data normalization

During limited proteolysis two types of peptide are formed. Fully tryptic peptides originate from trypsin cleavage alone, and semitryptic peptides result from PK cleavage followed by trypsin cleavage. We identified differences in accessibility between fractions with the following data analysis pipeline.

All peptide or protein intensities of a given LiP or tryptic control Spectronaut search were median normalized before exporting the data (global normalization). For the HU-induced stress response experiments, the peptide and/or protein intensities were median normalized before exporting the data. For the interface marker library, a separate Spectronaut search was conducted for every filter fraction and data were median normalized only within replicates of one fraction before exporting. The intensity values were based on Spectronaut normalized peak areas.

Further data processing was performed using the statistical software R (v.4.1.1) (R Foundation for Statistical Computing, http://www.R-project.org/). To account for differences in protein abundances across conditions, we corrected peptide intensities in condition *c* and replicate rep, ${I}_{{\mathrm{pep}},{c},{\mathrm{rep}}}$, by their corresponding protein intensities, ${I}_{{\mathrm{prot}},{c},{\mathrm{rep}}}$. For the interface marker library experiment, we further median normalized ${R}_{{\mathrm{pep}},{c},{\mathrm{rep}}}$ (equation 1), because the ratios significantly differed between filter fractions due to differences in protein abundances. This reflects that for most peptides, the ratio of peptide to protein stays constant across fractions and peptides for which this ratio changes across fractions pinpoint the location of a difference in accessibility between fractions and indicative of a PBI.

1 $${R}_{{\mathrm{pep}},c,{\mathrm{rep}}}=\,\frac{{I}_{{\mathrm{pep}},c,{\mathrm{rep}}}}{{I}_{{\mathrm{prot}},c,{\mathrm{rep}}}}$$

### Significance analysis

To detect peptides for which ${R}_{{\mathrm{pep}},{c},{\mathrm{rep}}}$ is significantly different between conditions (or filter fractions), we perform a one-way ANOVA test for all peptides detected in two or more conditions. We calculate the mean intensity of the peptide pep in condition *c* as:

2 $${\mathrm{Mean}}_{\mathrm{pep},c}=\frac{{\sum }_{{\mathrm{rep}}=1}^{{n}_{{\mathrm{pep}},c}}\,{R}_{{\mathrm{pep}},c,{\mathrm{rep}}}}{{n}_{{\mathrm{pep}},c}}$$

where ${n}_{\mathrm{pep},{c}}$ is the number of biological replicates (three or four) that a peptide was detected in condition *c*. Next, we propagate the error that results from the joint uncertainty in the peptide intensity and the protein intensity for every ${\mathrm{Mean}}_{\mathrm{pep},{c}}$ as:

3 $${\mathrm{s.d.}}_{\mathrm{pep},c}={\mathrm{Mean}}_{\mathrm{pep},c}\,\sqrt{{\left(\frac{{\rm{\sigma }}({I}_{{\mathrm{pep}},c})}{{\rm{\mu }}({I}_{{\mathrm{pep}},c})}\right)}^{2}+{\left(\frac{{\rm{\sigma }}({I}_{{\mathrm{prot}},c})}{{\rm{\mu }}({I}_{{\mathrm{prot}},c})}\right)}^{2}\,}$$

where *σ* denotes the standard deviation and *μ* the mean of the peptide pep and the corresponding protein prot intensities in condition *c*. The between group mean squared error is given by:

4 $${\mathrm{MS}}_{{\mathrm{between}},{\mathrm{pep}}}=\,\frac{\sum _{c}{n}_{{\mathrm{pep}},c}{\left({\mathrm{Mean}}_{{\mathrm{pep}},c}-\frac{\sum _{c}{\mathrm{Mean}}_{{\mathrm{pep}},c}}{{N}_{\mathrm{pep}}}\right)}^{2}\,}{{C}_{\mathrm{pep}}}$$

where *N*_pep_ is the number of times a peptide was measured across all conditions and *C*_pep_ the number of conditions it was detected in. The within group mean squared error is given by:

5 $${\mathrm{MS}}_{{\mathrm{within}},{\mathrm{pep}}}=\,\frac{\sum _{\mathrm{frac}}({n}_{{\mathrm{pep}},c}-1\,)\,{{\mathrm{s.d.}}_{{\mathrm{pep}},c}}^{2}\,}{{N}_{\mathrm{pep}}-\,{C}_{\mathrm{pep}}}$$

Finally, the *F* value is given by:

6 $${F}_{\mathrm{pep}}=\,\frac{{\mathrm{MS}}_{{\mathrm{between}},{\mathrm{pep}}}}{{\mathrm{MS}}_{{\mathrm{within}},{\mathrm{pep}}}}$$

The *F* value *F*_pep_ follows an *F* distribution with (${C}_{\mathrm{pep}}-1$, ${N}_{\mathrm{pep}}-\,{C}_{\mathrm{pep}}$) degrees of freedom. The *P* value is given by the probability of observing an event at least as extreme as the *F*_pep_ value. The *P* values are corrected for multiple testing with the Benjamini–Hochberg method and reported as *q* values^118^.

Multiple peptides can overlap because they map to the same protein regions because they originate from the same fully tryptic parent peptide. This can be caused by distinct PK cleavage sites since PK cleavage is a stochastic process or by missed cleavages. We group those peptides back to the longest identified fully tryptic parent and group the *q* value by its median over all peptides. We name those uniquely identified positions as FLiP markers.

### Marker classification

In the FLiP marker library, some proteins elute in lower molecular weight fractions than expected by their size. This can be due to the property of the protein itself or by cleavage events occurring in the cell or during the experimental procedure. Due to the nonstrict nature of the filter cutoffs, we classify proteins as eluting in the correct fraction if half of their molecular weight does not exceed the filter cutoff (for example an 80-kDa protein is allowed to elute in the 30-kDa fraction where all proteins are expected to be below 50 kDa, but not in the 10-kDa fraction where all proteins are expected to be below 30 kDa).

Proteins ending up in the wrong fraction can lead to significant changes detected by FLiP. We perform a Tukey honest significant difference test with adjustment for multiple testing (Benjamini–Hochberg) to assess the significance of a change between any two fractions. If a change is only significant when including the wrong molecular weight fraction, we classify the marker as low confident, high confident otherwise.

Also, we add information about previous evidence for a marker to be located at a known binding interface by annotating whether the marker peptide is overlapping with an InterPro domain annotated with the GO-terms ‘protein binding’ or ‘RNA binding’ or if its average distance to a binding interface in a PDB structure is <2.6 Å (see the section 'Distance of FLiP markers to PBIs of multimeric structures').

### Integrating FLiP marker library into LiP–MS datasets

A peptide detected in any LiP–MS dataset was classified as FLiP–MS marker peptide if it was overlapping by at least 50% with a peptide from the library.

### Domain-based GO-enrichment analysis

Protein domain GO-annotation on molecular function for all detected peptides was downloaded from the InterPro database^35^ (December 2021). A Fisher Exact test was computed to detect domains enriched in the FLiP library compared to all detected peptides.

### Disorder analysis

Protein disorder information for the yeast proteome was downloaded from UniProt^119^ (July 2022). If a peptide of the FLiP dataset was at least partially overlapping with a disordered region it was classified as disordered. The percentage of disordered peptides for all nonsignificant and significant peptides of the FLiP dataset was calculated. A Fisher Exact test was performed to test against the null hypothesis that significant FLiP peptides are not disordered.

### Distance of FLiP markers to PBIs of multimeric structures

We collected experimental structures associated with the relevant UniProt accession numbers^119^ as defined by SIFTS^120^ on 9 May 2022, through the SWISS-Model Repository API^121^. Analysis was performed on oligomers depicted as ‘biological assembly 1’. In case of multiple oligomers for the same UniProt accession number, we chose the structure with the most subunits (biggest), a structure at random (random) or the structure that best reflected the experimental data was considered. This left between 364 and 394 oligomeric structures for analysis depending on the selection method (Supplementary Table 2). Interface residues were identified by computing relative solvent accessibilities as described by Lee and Richards^122^ as implemented in OpenStructure^123^. Residues were defined as ‘interface’ if the change was above 25% when computed on the full oligomers as compared to single chains in isolation.

All FLiP peptides of a given protein were mapped to the corresponding PDB structure by sequence. FLiP peptides absent from the structure were discarded from the analysis. For all remaining peptides, distances (Cα carbon to Cα carbon) to these interface residues determined the labeling of the FLiP peptides. Each peptide residue was assigned the minimum average distance to any of the interface residues. If the distance was below the selected cutoff (2.6 or 0.3 Å), the peptide was labeled as interface associated (that is, positive). Whenever an oligomer contained several occurrences of the same peptide, a peptide was randomly selected for the labeling procedure. A ROC area under the curve (AUC) was used to determine whether the significance level of a peptide predicted the proximity to an oligomer interface. For the ROC analysis we classified marker peptides located at interfaces as true positives, marker peptides not located at interfaces as false positives, nonchanging peptides located at interfaces as false negatives and nonchanging peptides not located at interfaces as true negatives, resulting in AUCs of 0.54 (biggest), 0.54 (random) and 0.61 (best) when using 2.6 Å as a cutoff, and AUCs of 0.61 (biggest), 0.63 (random), 0.71 (best) at the more stringent 0.3-Å cutoff. The analysis was done in python v.3.7 and packages pickle5 v.0.0.11, matplotlib v.3.1.3 and sklearn v.0.22.1.

### AlphaFold3 predictions of heterodimeric protein complexes

We used network analysis (sections below) to extract heterodimeric protein complexes from the Complex Portal database, requiring that they had FLiP markers for both subunits and no previous structural characterization. Around 30 heterodimeric protein structures were predicted on the AlphaFold server (https://alphafoldserver.com/about) with seed 42, of which only two had a low predicted alignment error and are reported in this study.

### Calculating the distance of peptides to the protein surface

If FLiP markers are enriched in PBIs, they should be located closer to the protein surface compared to other LiP hits, since the latter can contain buried small-molecule binding sites. To test this hypothesis, we first defined the surface by the MSMS algorithm^124^ (MSMS v.2.5.7, get_surface, Bio.PDB.ResidueDepth v.1.83) with increasing probe radius. Increasing the radius will define buried sites as not located at the surface. The distance of a peptide to the surface was calculated with scipy.spatial.cKDTree (v.1.13.0) from the PK cleavage site for semitryptic and from the trypsin cleavage site for fully tryptic peptides.

### Acetylation analysis

For each comparison (wild-type control to wild-type HU, mutant control to mutant HU, wild-type control to mutant control, wild-type HU to mutant HU), a separate Spectronaut search with the respective sample files was performed with the spectral library containing acetylated peptides and imputation ‘Use Background Signal’. Each dataset was median normalized in Spectronaut (global normalization) before exporting the peptide and protein intensities separately. Differential abundance analysis was performed as described above.

### AP–MS analysis

High confidence interactors of Ada3 under control and HU-stressed conditions were determined separately by comparing the spectral counts of the FLAG-tagged to the nontagged samples in SAINT^125^. We identified 238 interactors of Ada3-FLAG by requiring a SAINT probability of 1 and that the protein was detected in four out of four replicates in the pull-down with a minimal average spectral count of 4.5. Intensities of high confidence interactors (SAINT = 1) for FLAG-tagged and nontagged samples under control and HU stress were normalized together by total area sums and only proteins detected in at least triplicates per condition were considered for downstream analysis. The ratios of prey to bait ${R}_{{\mathrm{prey}},{\mathrm{bait}},{\mathrm{treatment}}}$ was defined as follows, where ${{\mu }}(I)$ denotes the mean protein intensity of the respective protein in either the FLAG-tagged or nontagged cells under untreated or HU-treated conditions. In our case Ada3 is the bait.

7 $${R}_{{\mathrm{prey}},{\mathrm{bait}},{\mathrm{treatment}}}=\,\frac{\frac{{{\mu }}({I}_{{\mathrm{prey}},{\mathrm{FLAG}},{\mathrm{treatment}}})}{{{\mu }}({I}_{{\mathrm{prey}},{\mathrm{non}}-{\mathrm{FLAG}},{\mathrm{treatment}}})}}{\frac{{{\mu }}({I}_{{\mathrm{bait}},{\mathrm{FLAG}},{\mathrm{treatment}}})}{{{\mu }}({I}_{{\mathrm{bait}},{\mathrm{non}}-{\mathrm{FLAG}},{\mathrm{treatment}}})}}$$

We propagated the error of each ratio as:

8 $$\begin{array}{l}{\mathrm{s.d.}}_{{\mathrm{prey}},{\mathrm{bait}},{\mathrm{treatment}}}\\={R}_{{\mathrm{prey}},{\mathrm{bait}},{\mathrm{treatment}}}\,\sqrt{\begin{array}{c}{\left(\displaystyle\frac{{\rm{\sigma }}\left({I}_{{\mathrm{prey}},{\mathrm{FLAG}},{\mathrm{treatment}}}\right)}{{\rm{\mu }}\left({I}_{{\mathrm{prey}},{\mathrm{FLAG}},{\mathrm{treatment}}}\right)}\right)}^{2}+{\left(\displaystyle\frac{{\rm{\sigma }}\left({I}_{{\mathrm{prey}},{\mathrm{non}}-{\mathrm{FLAG}},{\mathrm{treatment}}}\right)}{{\rm{\mu }}\left({I}_{{\mathrm{prey}},{\mathrm{non}}-{\mathrm{FLAG}},{\mathrm{treatment}}}\right)}\right)}^{2}\,\\ +{\left(\displaystyle\frac{{\rm{\sigma }}({I}_{{\mathrm{bait}},{\mathrm{FLAG}},{\mathrm{treatment}}})}{{\rm{\mu }}({I}_{{\mathrm{bait}},{\mathrm{FLAG}},{\mathrm{treatment}}})}\right)}^{2}+{\left(\displaystyle\frac{{\rm{\sigma }}({I}_{{\mathrm{bait}},{\mathrm{non}}-{\mathrm{FLAG}},{\mathrm{treatment}}})}{{\rm{\mu }}({I}_{{\mathrm{bait}},{\mathrm{non}}-{\mathrm{FLAG}},{\mathrm{treatment}}})}\right)}^{2}\end{array}}\end{array}$$

where *σ* denotes the standard deviation and *μ* the mean of the given protein intensity. We calculate the *t*-statistic as:

9 $${t}_{{\mathrm{prey}}}=\frac{{R}_{{\mathrm{prey}},{\mathrm{bait}},{\mathrm{HU}}-{\mathrm{treated}}}-{R}_{{\mathrm{prey}},{\mathrm{bait}},{\mathrm{untreated}}}}{\sqrt{\frac{{{\mathrm{s.d.}}_{{\mathrm{prey}},{\mathrm{bait}},{\mathrm{HU}}-{\mathrm{treated}}}}^{2}+{{\mathrm{s.d.}}_{{\mathrm{prey}},{\mathrm{bait}},{\mathrm{untreated}}}}^{2}}{3}}\,}$$

The *t*-statistic follows a Student’s *t* distribution with four degrees of freedom. The *P* value is given by the probability of observing an event at least as extreme as the *t* value *P*(*T* > *t*_prey_). The log_2_ fold change is given by the ratio log_2_(*R*_prey,bait,HU-treated_/*R*_prey,bait,untreated_).

### Network analysis

All 586 proteins with at least one significantly changing FLiP marker in the HU-induced DNA replication stress screen were projected on a PPI network based on Complex Portal^40^ (16 March 2021). Out of those 586 proteins, only 206 proteins are part of the network. Note that we used this database as ground truth because complexes are defined with high stringency, based on experimental physical interaction data, but in principle another database could also be used. The network was analyzed in R (v.4.1.1) using igraph (v.1.2.6). The network was filtered for self-loops and redundancies. All edges were considered undirected and unweighted. First, the network was propagated from the proteins with interface marker hits in the HU stress screen using the PageRank algorithms with a dampening factor of 0.9 (ref. ^126^). The most important part of the network was selected based on scoring in the top 40% of this propagation resulting in a network consisting of 722 proteins. We chose the dampening factor of 0.9 and the 40% PageRank score cutoff as it yielded a network that was neither too sparse nor too dense, making it possible to identify protein complexes. We then used the walktrap algorithm^127^ with four steps to cluster the resulting network, again choosing the number of steps to obtain good separation between clusters. Finally, we selected clusters and/or complexes with at least four subunits, aiming to filter out dimeric complexes that are typically less well annotated. This is the final network that we report, containing 607 proteins with 56 complexes. Note that that there is no single optimal threshold for any of these steps, and a user may choose them differently depending on their systems and the goal of the experiment. We named the resulting clusters based on the protein complex from the Complex Portal database with the highest number of changing FLiP markers present in that cluster, reasoning that the higher the number of marker changes in a given protein complex, the more likely it is that this specific complex is rearranging. Because many protein complexes share subunits, we sometimes provide more than one name for a cluster if all changing markers are part of multiple complexes (for example, DNA-directed RNA polymerase I, II or III complexes or the INO80–Swr1–Nu4A complex). For the comparison of the response in PPI changes of wild type and the Gcn5 catalytic dead cells, the data were reduced to only contain protein regions detected in both datasets.

### Data visualization

Data were visualized using the statistical software R (v.4.1.1) with the packages ggplot2 (v.3.3.5), RColorBrewer (v.1.1.2), ComplexHeatmap (v.2.8.0), igraph (v.1.2.6) and EnhancedVolcano (v.1.18.0). Peak groups and protein sequence localization plots were visualized using Microsoft Excel (v.16.0.5332.1000).

### Protein structure visualization

Protein structures were retrieved from the PDB^128^ and visualized in PyMol (v.2.4.1). The peptide sequences were aligned to the PDB sequence and colored according to their classification.

### SDS–PAGE and immunoblotting of C-terminally GFP-tagged Spt7

BY4741 MATa his3Δ1 leu2Δ0 ura3Δ0 met15Δ0 spt7-GFP:HIS3 from the yeast GFP collection^113^ was grown and harvested in a single replicate under control and HU stress as described above. Protein concentration was adjusted to 3 mg ml^−1^ and 40 μl were mixed with 360 μl 5% (w/v) sucrose stock (5% (w/v) sucrose, 150 mM NaCl, 1 mM MgCl_2_, 100 mM HEPES pH 7.4, 2× Roche Complete protease inhibitor). Proteins were precipitated on ice for 1 h by adding trichloroacetic acid to a final concentration of 9% (v/v), followed by centrifugation at 21,000*g* for 10 min at 4 °C. The supernatant was discarded, and the protein pellets washed twice with ice-cold acetone (500 μl per sample, spin in between washes at 21,000*g* for 10 min at 4 °C). Pellets were air-dried for 5–10 min after removing residual acetone and resuspended in LDS NuPage Sample buffer (NP0007, Thermo Fisher) containing 137.5 mM DTT and 312.5 mM Tris base. Samples were heated at 95 °C for 10 min under constant agitation (Eppendorf thermomixer, 1,500 rpm) before separation on NuPage 4–12% Bis/Tris gradient gels. Proteins were then transferred to a PVDF membrane and membranes blocked with 5% milk powder (w/v) in Tris-buffer saline (TBS) + 0.1% Tween for 1 h before incubation with an anti-GFP antibody (Roche, 11814460001, 1:1,000 in 2% BSA/TBS + 0.1% Tween) under constant agitation for 16 h at 4 °C. Membranes were rinsed with TBS + 0.1% Tween 3× 5 min and incubated with HRP-conjugated secondary antibody (Invitrogen, A16078, 1:10,000 in 5% milk powder (w/v) in TBS + 0.1% Tween) for 1 h and rinsed with TBS + 0.1% Tween 3× 5 min before application of ECL substrate (ECL Clarity) and image acquisition (Vilber Fusion FX).

### Spot growth assay

Yeast cells were grown in liquid culture for >16 hours to an optical density (OD_600_) between 0.6 0.8. These cultures were diluted to a starting OD_600_ of 0.1, then fivefold serial dilutions were spotted onto different media in 2-µl amounts and grown at 30 °C for 4 days. Plates comprised either: synthetic complete medium and 2% glucose, or synthetic complete medium, 2% glucose and 100 mM HU (Sigma-Aldrich). Images were acquired on a Bio-Rad Gel Doc XL+ (Bio-Rad) using transmitted white light.

### Fluorescence microscopy

Cells expressing Dcp2-GFP from the endogenous locus were imaged using a Nikon Ti-E Eclipse (Nikon Instruments), controlled by Micro-Manager v.1.4.23 software^129^, with a Plan Apo ×60 1.4 NA objective. A CoolLED pE-300 (CoolLED) light source was used for fluorescence illumination. To rapidly assess P-body formation in different conditions, a 1-ml sample of cells was concentrated by centrifugation (600*g*, 2 min) and visualized immediately by wet mount.

### Time-lapse microscopy and analysis

Cells were grown for >16 h to OD_600_ = 0.6–0.8, diluted to OD_600_ = 0.2, then loaded onto a CellASIC ONIX Y04 microfluidic plate (Merck Millipore), which was mounted on the microscope described above, housed in a temperature-controlled incubator set to 30 °C. Cells were grown under normal conditions (synthetic complete medium, 2% glucose) for 3 h, switched to the stress condition (synthetic complete medium, 2% glucose, 200 mM HU) for 3 h, then switched back to normal conditions for 6 h. Media switching was controlled using CellASIC ONIX software. The flow rate was maintained at 3.5 psi throughout. Brightfield images were acquired every 15 min and fluorescence images every 30 min, in a single focal plane. Images were acquired more rapidly (5-min intervals for both channels), for a 40-min period centered on the switch to stress conditions. Time-lapse videos were analyzed by extracting the mean and standard deviation of fluorescence pixel intensities within each cell at each timepoint, based on masks generated by segmenting cells in the brightfield channel, using YeaZ software^130^. The averaged single-cell quantifications of Dcp2-mNG dispersion index were calculated as the variance in pixel intensities within a cell divided by mean pixel intensity within the same cell. For image display, 16-bit raw microscopy images were background subtracted in Fiji using a 200 pixel rolling ball radius. They were linearly scaled in Fiji, converted to 8-bit, and displayed in greyscale. In each figure, scaling was identical between images representing different time points and/or conditions, to allow accurate comparison of the signals. All quantifications were carried out on 16-bit images after background subtraction (200-pixel rolling ball radius).

### Reporting summary

Further information on research design is available in the Nature Portfolio Reporting Summary linked to this article.

## Online content

Any methods, additional references, Nature Portfolio reporting summaries, source data, extended data, supplementary information, acknowledgements, peer review information; details of author contributions and competing interests; and statements of data and code availability are available at 10.1038/s41587-024-02432-8.

## Supplementary information

Supplementary Information Listing and short description of all supplementary tables. Supplementary Table 2 and Figs 1–6.

Reporting Summary

Supplementary Table 1 Workbook with Supplementary Tables 1 and 3–6.

## Source data

Source Data Fig. 4 Unprocessed western blot.

## Data Availability

The mass spectrometry proteomics data have been deposited to the ProteomeXchange Consortium via the PRIDE^131^ partner repository with the dataset identifier PXD055566. Source data are provided with this paper.

## Code availability

All code is available on GitHub (https://github.com/cmarulli/FLiP-MS)^117^.
