# Supplementary material for: Global profiling of protein complex dynamics with an experimental library of protein interaction markers
Source: Nat Biotechnol. 2024 Oct 16;43(9):1562–76. doi: 10.1038/s41587-024-02432-8 (PMC12440823; doi:10.1038/s41587-024-02432-8)
Supplement: Supplementary file 1 — Listing and short description of all supplementary tables. Supplementary Table 2 and Figs 1–6. [file 41587_2024_2432_MOESM1_ESM.pdf]

nature biotechnology

Supplementary information

https://doi.org/10.1038/s41587-024-02432-8

Global profiling of protein complex dynamics with an experimental library of protein interaction markers

In the format provided by the authors and unedited

Supplementary Tables

All tables not displayed here can be accessed via the Excel workbook in the supplementary materials.

Table ST1: FLiP library.

PDB Selection	Proteins	PDBs	Mapped Peptides	Cutoff (Å)	Peptides at Interface	TPs	FPs	AUC
Biggest	417	373	6546	2.6	1358	687	2237	0.54
Biggest	417	373	6546	0.3	160	92	2832	0.61
Random	443	394	6592	2.6	1349	672	2234	0.54
Random	443	364	6592	0.3	143	85	2821	0.63
Best	443	365	6546	2.6	1356	811	2057	0.61
Best	443	365	6428	0.3	179	140	2655	0.71

Table ST2 Summary table for peptide mapping using different ground truth datasets. Shown are: the number of proteins from the FLiP-MS dataset with at least one peptide mapping to the selected PDB structures (Proteins); the number of PDB structures selected for the proteins in the FLiP-MS dataset (PDBs); the number of peptides from the FLiP-MS dataset mapped to the selected PDBs (Mapped Peptides); the distance used as a cutoff to define a peptide as being located at the interface (Cutoff); the number of peptides classified as being located at an interface (Peptides at Interface); the number of true positives (TPs) and false positives (FPs); the area under the ROC curve (AUC).

Table ST3: Protein abundance changes in wild type cells between HU-stress and control.

Table ST4: Protein structural changes in wild type cells between HU-stress and control.

Table ST5: Protein complexes likely to undergo changes in assembly in wild type cells upon HU-stress.

Table ST6: Changes in acetylated peptides in wild type cells between HU-stress and control.

Table ST7: Protein abundance changes in Gcn5 catalytic dead cells between HU-stress and control.

Table ST8: Protein structural changes in Gcn5 catalytic dead cells between HU-stress and control.

Table ST9: Comparison of protein complexes likely to undergo changes in assembly upon HU-stress between wild type and Gcn5 catalytic dead cells.

Table ST10: DIA acquisition windows.

Supplementary Figures

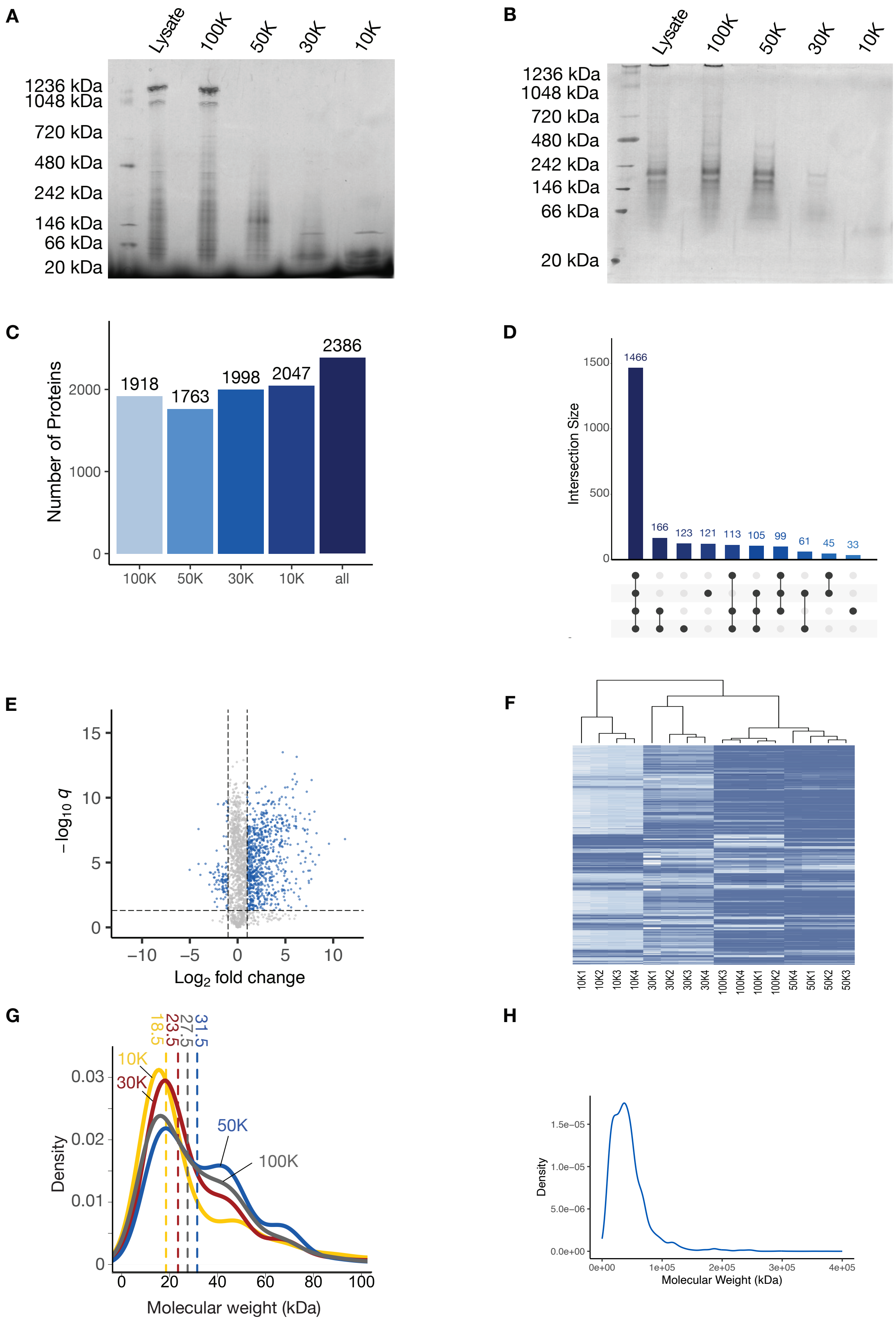

Figure S1 Separation of proteins and protein assemblies by serial ultrafiltration. **A)** 7% and **B)** 3-8% NuPAGE gel of 10 µg protein from the lysate, 100K, 50K, 30K and 10K fraction**.** Both gels are single replicate experiments with comparable results. **C)** Number of proteins identified in at least triplicates in the four filter fractions and across all fractions processed by quantitative mass spectrometry. **D)** Number of proteins identified in the indicated intersections of filter fractions. **E)** Volcano plot of differential abundance of proteins between fractions for all proteins identified in at least two fractions. If a protein was identified in more than two fractions, the fold change shown corresponds to the maximal fold change between any fractions. P-values were determined by ANOVA test (one-way) between all filter fractions where the protein was detected and adjusted for multiple testing (Benjamini-Hochberg). **F)** Hierarchically clustered heat map of the identified protein abundances for all filter fractions and replicates. **G)** Density distribution of the molecular weight of identified proteins in the indicated filter fractions after serial ultrafiltration. Dotted lines indicate the intensity weighted median MWs of every distribution. **H)** Intensity-weighted density distribution of the molecular weight of the yeast proteins detected in a full lysate by mass-spectrometry.

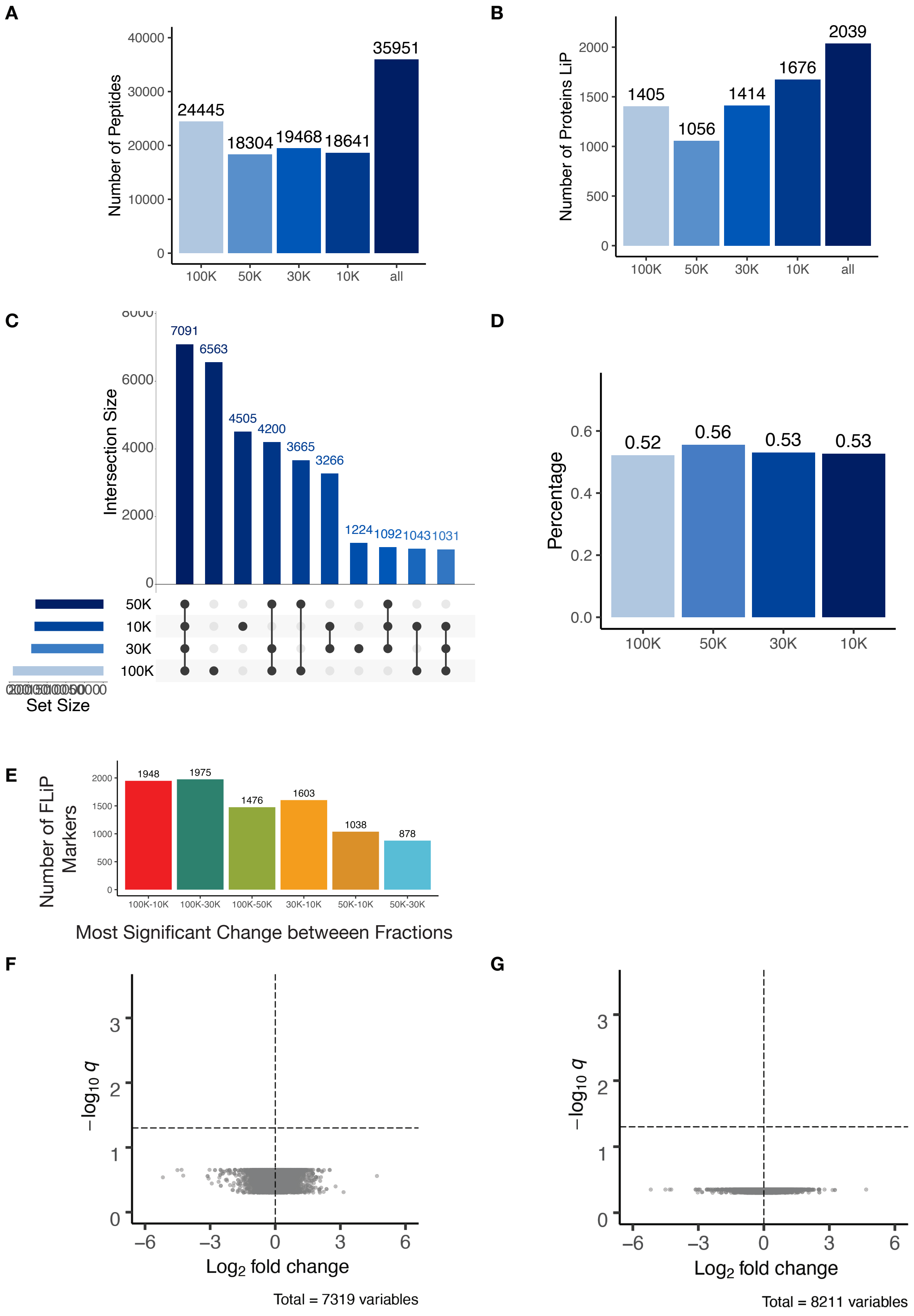

A
Number of Peptides
24445
18304
19468
18641
35951
100K
50K
30K
10K
all
B
Number of Proteins LiP
1405
1056
1414
1676
2039
100K
50K
30K
10K
all
C
Intersection Size
7091
6563
4505
4200
3665
3266
1224
1092
1043
1031
50K
10K
30K
100K
Set Size
D
Percentage
0.52
0.56
0.53
0.53
100K
50K
30K
10K
E
Number of FLiP Markers
1948
1975
1476
1603
1038
878
100K-10K
100K-30K
100K-50K
30K-10K
50K-10K
50K-30K
Most Significant Change betweeen Fractions
F
$-\log_{10} q$
Log_2 fold change
Total = 7319 variables
G
$-\log_{10} q$
Log_2 fold change
Total = 8211 variables

Figure S2 Assessments of the FLiP-MS dataset. **A)** Number of peptides identified in at least triplicates in the four filter fractions and across all fractions processed by LiP-MS. **B)** Number of proteins identified in at least triplicates in the four filter fractions and across all fractions processed by LiP-MS. **C)** Number of peptides identified in the indicated intersections of filter fractions processed by LiP-MS. **D)** Percentage of total intensity signal from semi-tryptic peptides identified in the filter fractions. **E)** Fractions between which the most significant change for a FLiP marker was observed. **F)** Volcano plot showing differential abundance of LiP-MS peptides in a fresh lysate and lysate kept on ice for a duration corresponding to filtration time. **G)** Volcano plot showing differential abundance analysis of a LiP-MS experiment comparing fresh lysate and merged filter fractions.

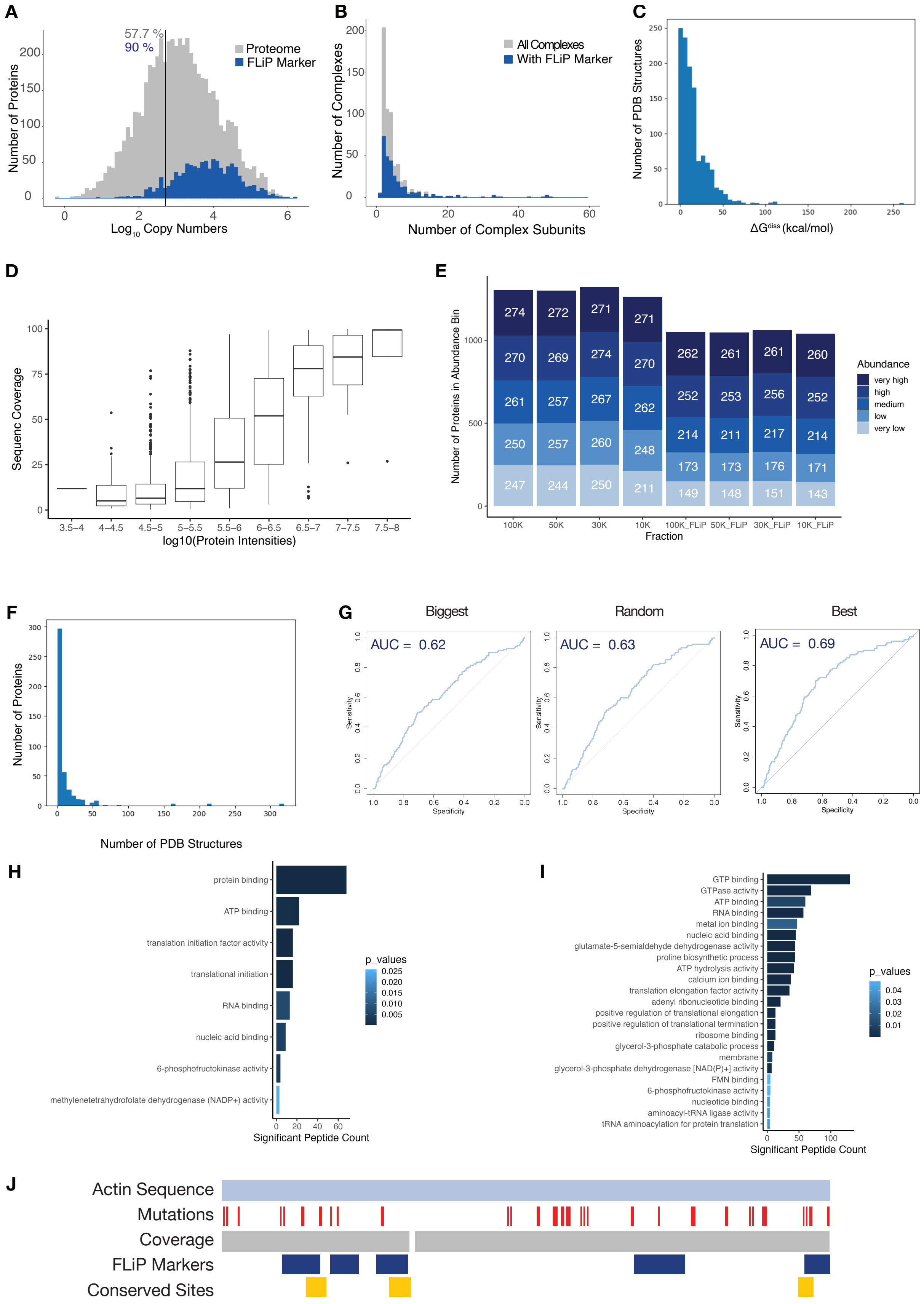
A
57.7 %
90 %
Proteome
FLiP Marker
Number of Proteins
Log10 Copy Numbers
B
All Complexes
With FLiP Marker
Number of Complexes
Number of Complex Subunits
C
Number of PDB Structures
ΔGdiss (kcal/mol)
D
Sequenc Coverage
3.5–4
4–4.5
4.5–5
5–5.5
5.5–6
6–6.5
6.5–7
7–7.5
7.5–8
log10(Protein Intensities)
E
Number of Proteins in Abundance Bin
274
272
271
271
270
269
274
270
262
261
261
257
267
262
252
253
256
252
250
257
260
248
214
211
217
214
247
244
250
211
173
173
176
171
149
148
151
143
100K
50K
30K
10K
100K_FLiP
50K_FLiP
30K_FLiP
10K_FLiP
Fraction
Abundance
very high
high
medium
low
very low
F
Number of Proteins
Number of PDB Structures
G
Biggest
Random
Best
AUC = 0.62
AUC = 0.63
AUC = 0.69
Sensitivity
Specificity
H
protein binding
ATP binding
translation initiation factor activity
translational initiation
RNA binding
nucleic acid binding
6-phosphofructokinase activity
methylenetetrahydrofolate dehydrogenase (NADP+) activity
p_values
Significant Peptide Count
I
GTP binding
GTPase activity
ATP binding
RNA binding
metal ion binding
nucleic acid binding
glutamate-5-semialdehyde dehydrogenase activity
proline biosynthetic process
ATP hydrolysis activity
calcium ion binding
translation elongation factor activity
adenyl ribonucleotide binding
positive regulation of translational elongation
positive regulation of translational termination
ribosome binding
glycerol-3-phosphate catabolic process
membrane
glycerol-3-phosphate dehydrogenase [NAD(P)+] activity
FMN binding
6-phosphofructokinase activity
nucleotide binding
aminoacyl-tRNA ligase activity
tRNA aminoacylation for protein translation
p_values
Significant Peptide Count
J
Actin Sequence
Mutations
Coverage
FLiP Markers
Conserved Sites

Figure S3 Characteristics of FLiP markers and their mapping to PBIs. **A)** Distribution of protein copy numbers per cell of the whole proteome (grey) and proteins corresponding to significantly changing FLiP-MS peptides (blue) [32]. **B)** Histogram showing the distribution of complex sizes (exact or minimal reported number of subunits in Complex Portal [40]) for all complexes in the Complex Portal database as well as the subset of complexes with a FLiP marker. **C)** PISA dissociation energy distribution of protein complexes with a PDB structure detected in the FLiP-MS dataset. PISA energies were downloaded from the PDBePISA database (https://www.ebi.ac.uk/pdbe/pisa/). **D)** Boxplots of sequence coverage depending on binned mean protein intensities (n = 1-479 proteins). The box displays the upper and lower quartile of the distribution with the middle line indication the median. **E)** Barplot showing the number of proteins detected (left four bars) and the number of proteins with at least one changing FLiP marker (right four bars) in 5 equally sized abundance bins (274 proteins per bin). **F)** Distribution of the number of PDB structures available for a given protein. **G)** ROC curves assessing mapping of marker peptides to known PBIs. A peptide was defined as at the interface if it has a 100% sequence overlap with the interface. **H)** InterPro domain-based GO-enrichment analysis of FPs from proteins only having FPs in the PDB analysis (Fisher-exact test, one-sided, $p < 0.05$). **I)** InterPro domain-based GO-enrichment analysis of FPs from proteins having both FPs and TPs in the PDB analysis (Fisher-exact test, one-sided, $p < 0.05$). **J)** Mutations in the IMEx database known to disrupt actin interactions. The annotated mutations are shown in red, the detected peptides in light grey, the FLiP markers in dark blue, and the conserved sites in yellow, all mapped along the linear actin protein sequence.

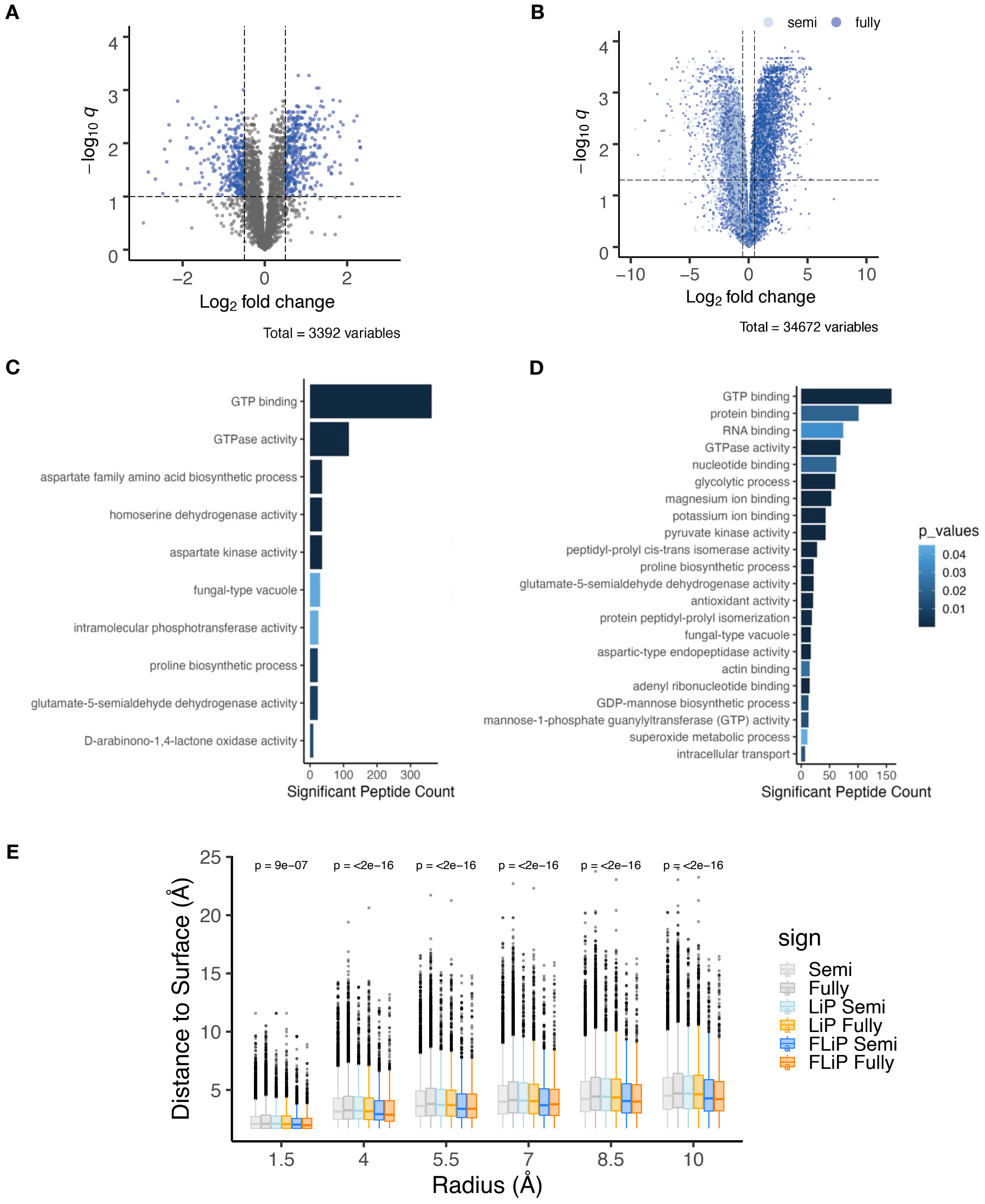
A
B
semi
fully
$-\log_{10} q$
Log2 fold change
Total = 3392 variables
Total = 34672 variables
C
GTP binding
GTPase activity
aspartate family amino acid biosynthetic process
homoserine dehydrogenase activity
aspartate kinase activity
fungal-type vacuole
intramolecular phosphotransferase activity
proline biosynthetic process
glutamate-5-semialdehyde dehydrogenase activity
D-arabinono-1,4-lactone oxidase activity
Significant Peptide Count
D
GTP binding
protein binding
RNA binding
GTPase activity
nucleotide binding
glycolytic process
magnesium ion binding
potassium ion binding
pyruvate kinase activity
peptidyl-prolyl cis-trans isomerase activity
proline biosynthetic process
glutamate-5-semialdehyde dehydrogenase activity
antioxidant activity
protein peptidyl-prolyl isomerization
fungal-type vacuole
aspartic-type endopeptidase activity
actin binding
adenyl ribonucleotide binding
GDP-mannose biosynthetic process
mannose-1-phosphate guanylyltransferase (GTP) activity
superoxide metabolic process
intracellular transport
Significant Peptide Count
p_values
0.04
0.03
0.02
0.01
E
p = 9e−07
p = <2e−16
p = <2e−16
p = <2e−16
p = <2e−16
p = <2e−16
Distance to Surface (Å)
Radius (Å)
sign
Semi
Fully
LiP Semi
LiP Fully
FLiP Semi
FLiP Fully

Figure S4 FLiP-MS analysis of wild-type yeast cells grown under HU-stress vs. control condition. **A)** Volcano plot showing differential protein abundance in HU-stressed cells compared to control (untreated) conditions. **B)** Volcano plot showing differential abundance of peptides from a LiP-MS experiment, normalized by protein abundance, in HU-stressed cells compared to control (untreated) conditions. Semi-tryptic peptides in light blue, fully tryptic peptides in dark blue. **C)** InterPro domain-based GO-enrichment analysis of the significant LiP-MS hits comparing wild type yeast cells grown under control and HU-induced replication stress (Fisher-exact test, one-sided, $p < 0.05$). **D)** InterPro domain-based GO-enrichment analysis of the significant LiP-MS hits comparing wild type yeast cells grown under control and HU-induced replication stress hits shortlisted with FLiP marker library (Fisher-exact test, one-sided, $p < 0.05$). **E)** Distance to the surface of non-changing semi-tryptic (Semi), non-changing fully-tryptic (Fully), changing semi-tryptic LiP peptides (LiP semi), changing fully-tryptic LiP peptides (LiP Fully), changing semi-tryptic FLiP marker peptides (FLiP Semi) and changing fully-tryptic FLiP marker peptides (FLiP Fully) (n = 1,154 – 11,887 peptides per group). The surface is defined by the rolling-ball algorithm and the distance is calculated for increasing radii of the ball. Differences between groups are calculated with an ANOVA (one-way, $p = 2^{-16}$–9^{-7}). The box displays the upper and lower quartile of the distribution with the middle line indication the median.

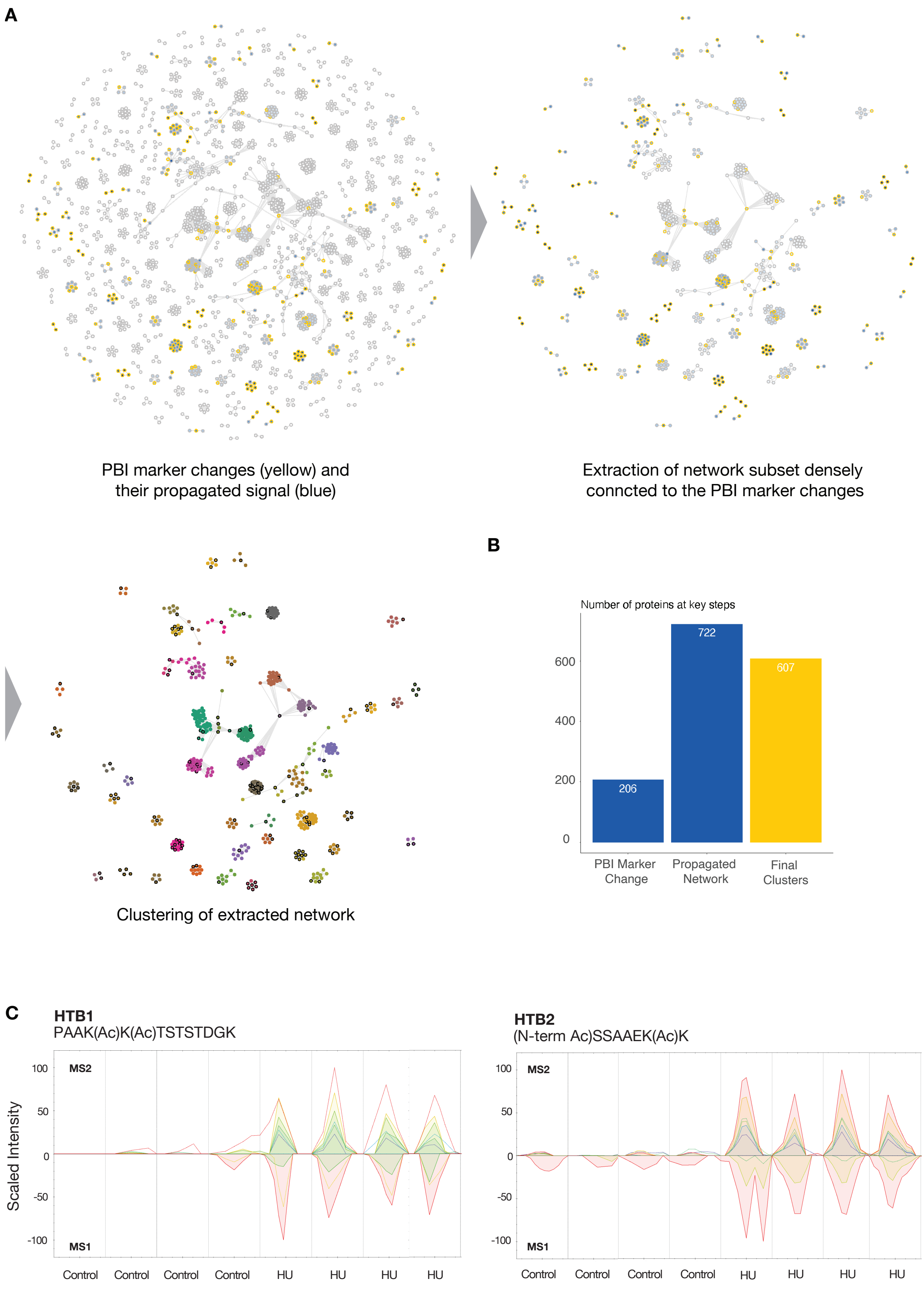
A
PBI marker changes (yellow) and their propagated signal (blue)
Extraction of network subset densely conncted to the PBI marker changes
Clustering of extracted network
B
Number of proteins at key steps
600
400
200
0
206
722
607
PBI Marker Change
Propagated Network
Final Clusters
C
HTB1
PAAK(Ac)K(Ac)TSTSTDGK
HTB2
(N-term Ac)SSAAEK(Ac)K
Scaled Intensity
100
50
0
-50
-100
MS2
MS1
Control
Control
Control
Control
HU
HU
HU
HU

Figure S5 Network analysis and acetylation changes upon HU-stress. **A)** Network representation of the results at crucial steps of the data analysis pipeline: (1) Projection of the hits onto a protein-protein interaction network and scoring of nodes with PageRank (2) Extracting nodes with PageRank scores in the top 40% (3) Clustering the resulting network with a minimal cluster size of four nodes. **B)** Bar diagram showing the number of proteins found to have changes in interaction (left), number of proteins extracted due to their PageRank score (middle), and number of proteins found in the regions rich in PPI changes (right). **C)** MS2 (upper part) and MS1 (lower part) peak groups of acetylated peptides of histone HTB1 and HTB2 for all replicates in control and under HU stress. The acetylated peptide of HTB2 corresponds exactly to the one identified as Gcn5 target in a previous acetylome profiling study, the HTB1 peptide was not identified in that study [82].

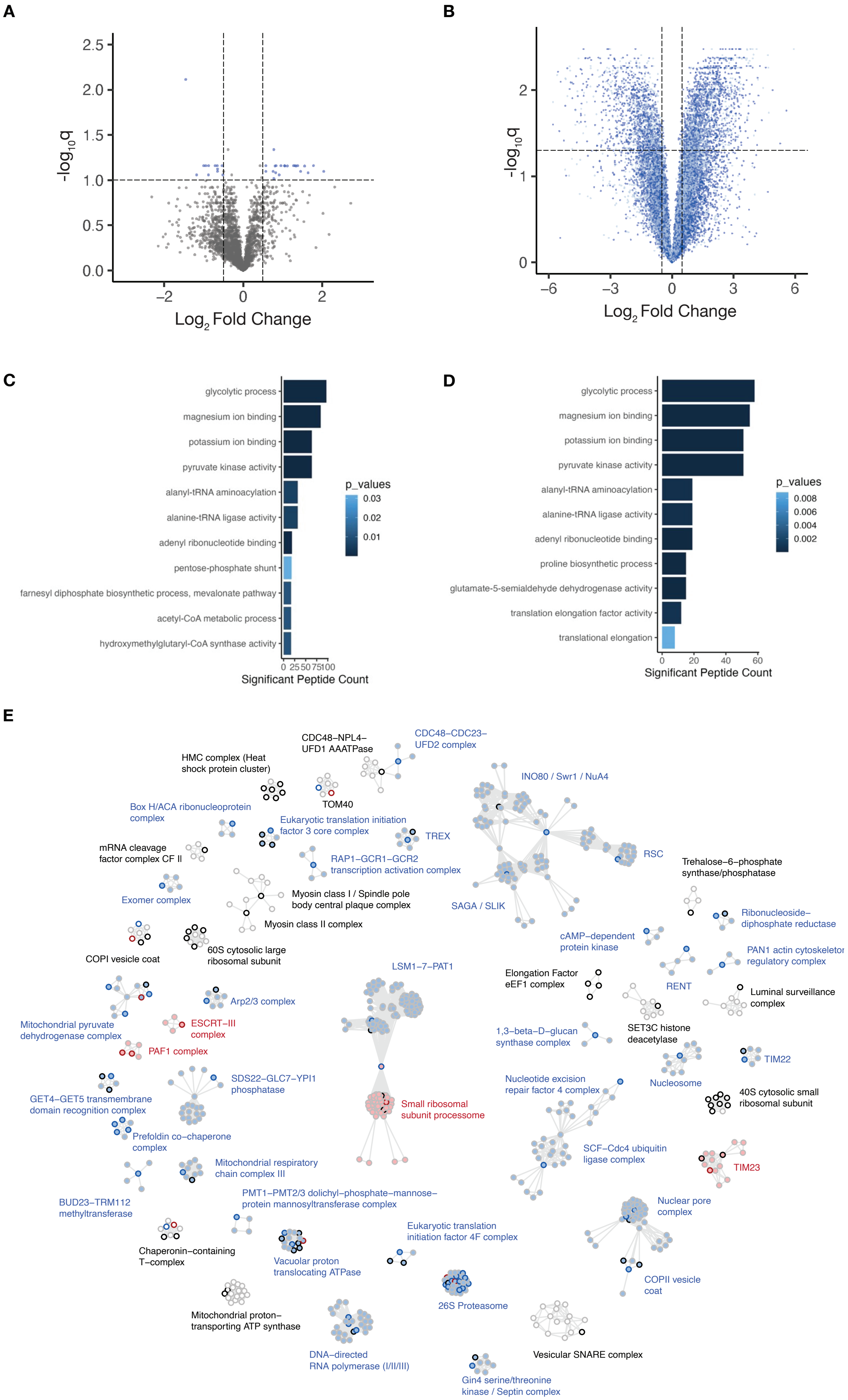
A
2.5
2.0
1.5
1.0
0.5
0.0
-log10q
−2
0
2
Log2 Fold Change
B
2
1
0
-log10q
−6
−3
0
3
6
Log2 Fold Change
C
glycolytic process
magnesium ion binding
potassium ion binding
pyruvate kinase activity
alanyl-tRNA aminoacylation
alanine-tRNA ligase activity
adenyl ribonucleotide binding
pentose-phosphate shunt
farnesyl diphosphate biosynthetic process, mevalonate pathway
acetyl-CoA metabolic process
hydroxymethylglutaryl-CoA synthase activity
p_values
0.03
0.02
0.01
0 255075100
Significant Peptide Count
D
glycolytic process
magnesium ion binding
potassium ion binding
pyruvate kinase activity
alanyl-tRNA aminoacylation
alanine-tRNA ligase activity
adenyl ribonucleotide binding
proline biosynthetic process
glutamate-5-semialdehyde dehydrogenase activity
translation elongation factor activity
translational elongation
p_values
0.008
0.006
0.004
0.002
0
20
40
60
Significant Peptide Count
E
CDC48–NPL4–UFD1 AAATPase
CDC48–CDC23–UFD2 complex
HMC complex (Heat shock protein cluster)
INO80 / Swr1 / NuA4
TOM40
Box H/ACA ribonucleoprotein complex
Eukaryotic translation initiation factor 3 core complex
TREX
mRNA cleavage factor complex CF II
RAP1–GCR1–GCR2 transcription activation complex
RSC
Trehalose−6−phosphate synthase/phosphatase
Exomer complex
Myosin class I / Spindle pole body central plaque complex
SAGA / SLIK
Ribonucleoside−diphosphate reductase
Myosin class II complex
cAMP−dependent protein kinase
COPI vesicle coat
60S cytosolic large ribosomal subunit
PAN1 actin cytoskeletor regulatory complex
LSM1−7−PAT1
Elongation Factor eEF1 complex
RENT
Luminal surveillance complex
Arp2/3 complex
Mitochondrial pyruvate dehydrogenase complex
ESCRT−III complex
1,3−beta−D−glucan synthase complex
SET3C histone deacetylase
PAF1 complex
TIM22
SDS22−GLC7−YPI1 phosphatase
Nucleotide excision repair factor 4 complex
Nucleosome
40S cytosolic small ribosomal subunit
GET4−GET5 transmembrane domain recognition complex
Small ribosomal subunit processome
Prefoldin co−chaperone complex
SCF−Cdc4 ubiquitin ligase complex
Mitochondrial respiratory chain complex III
TIM23
BUD23−TRM112 methyltransferase
PMT1−PMT2/3 dolichyl−phosphate−mannose−protein mannosyltransferase complex
Nuclear pore complex
Eukaryotic translation initiation factor 4F complex
Chaperonin−containing T−complex
Vacuolar proton translocating ATPase
COPII vesicle coat
26S Proteasome
Mitochondrial proton−transporting ATP synthase
DNA−directed RNA polymerase (I/II/III)
Vesicular SNARE complex
Gin4 serine/threonine kinase / Septin complex

F

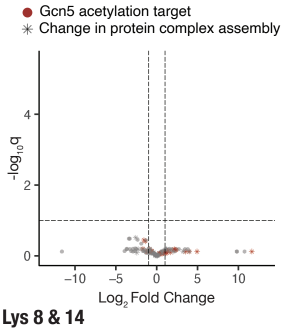

Gcn5 acetylation target
Change in protein complex assembly
-log10q
Log2 Fold Change

G

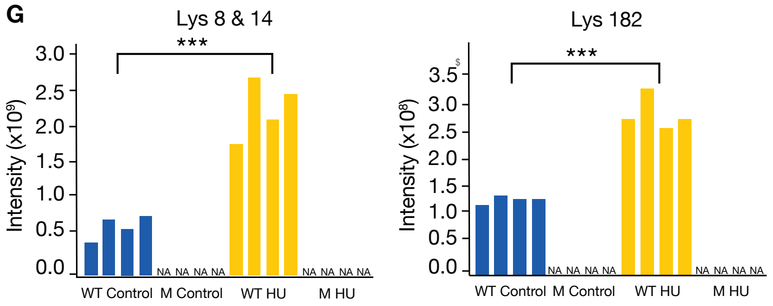

Lys 8 & 14
***
Intensity (x10⁹)
WT Control
M Control
WT HU
M HU
NA
Lys 182
***
Intensity (x10⁸)
WT Control
M Control
WT HU
M HU
NA

H

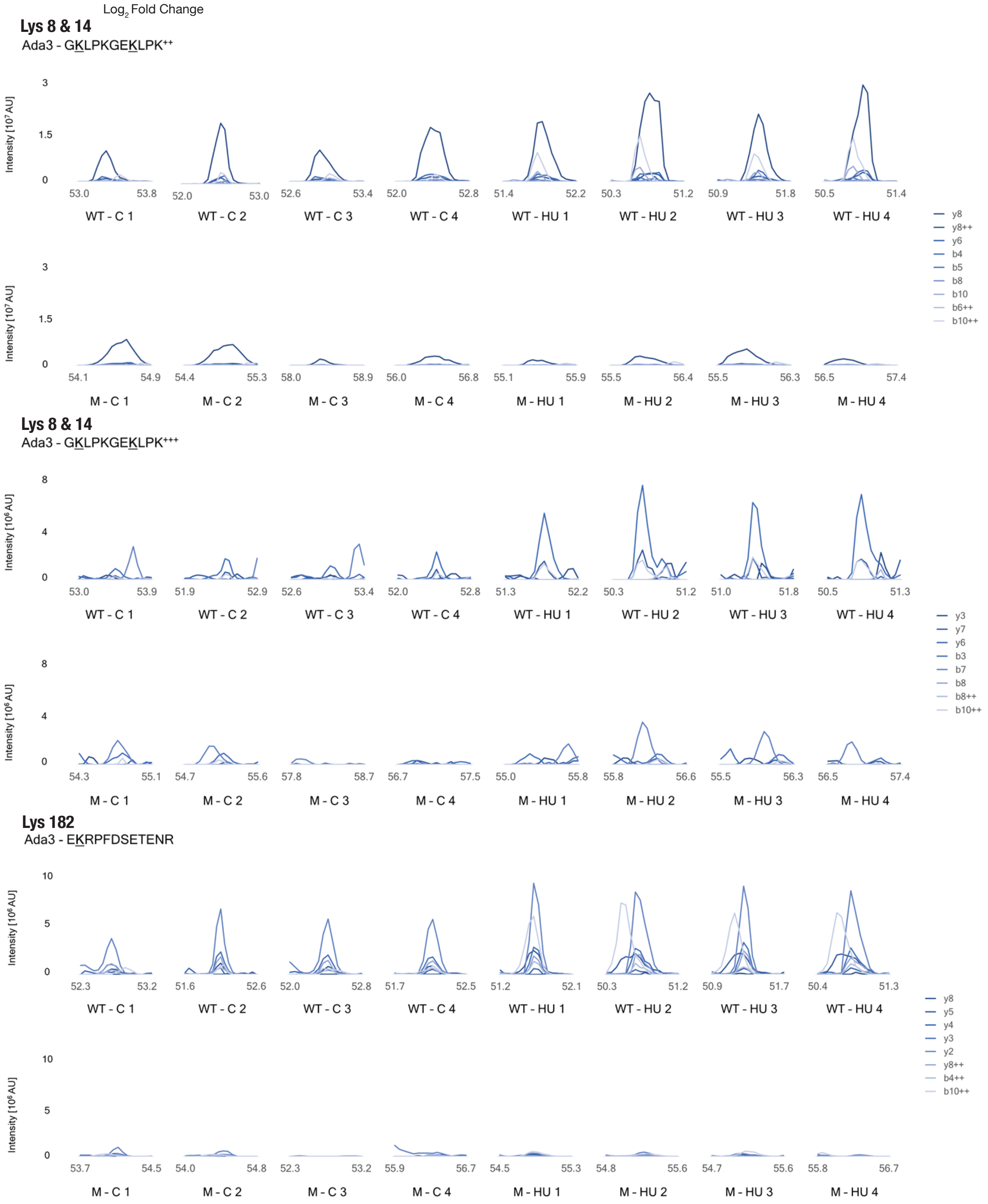

Lys 8 & 14
Ada3 - GKLPKGEKLPK++
Intensity [10⁷ AU]
WT - C 1
WT - C 2
WT - C 3
WT - C 4
WT - HU 1
WT - HU 2
WT - HU 3
WT - HU 4
M - C 1
M - C 2
M - C 3
M - C 4
M - HU 1
M - HU 2
M - HU 3
M - HU 4
y8
y8++
y6
b4
b5
b8
b10
b6++
b10++
Lys 8 & 14
Ada3 - GKLPKGEKLPK+++
Intensity [10⁶ AU]
y3
y7
y6
b3
b7
b8
b8++
b10++
Lys 182
Ada3 - EKRPFDSETENR
Intensity [10⁶ AU]
y8
y5
y4
y3
y2
y8++
b4++
b10++

Figure S6 FLiP-MS analysis of Gcn5 mutant cells grown under HU-stress vs. control condition and acetylation changes of Ada3. **A)** Volcano plot showing differential protein abundance in HU-stressed Gcn5 catalytic dead cells compared to control (untreated) conditions. **B)** Volcano plot showing differential abundance of peptides from a LiP-MS experiment, normalized by protein abundance, in HU-stressed Gcn5 catalytic dead cells compared to control (untreated) conditions. Semi-tryptic peptides in light blue, fully tryptic peptides in dark blue. **C)** InterPro domain-based GO-enrichment analysis of the significant LiP-MS hits comparing Gcn5 mutant cells grown under control and HU-induced replication stress (Fisher-exact test, one-sided, $p < 0.05$). **D)** InterPro domain-based GO-enrichment analysis of the significant LiP-MS hits comparing Gcn5 mutant cells grown under control and HU-induced replication stress hits shortlisted with FLiP marker library (Fisher-exact test, one-sided, $p < 0.05$). **E)** Protein complexes suggested to change assembly state under DNA replication stress in the wild type and Gcn5 catalytic dead cells. Changing FLiP markers between HU-stress and control are indicated by circles colored black if the marker changes in both the wild type and the Gcn5 catalytic dead cells, blue if the marker only changes in wild type cells, and red if the marker only changes in mutant cells. Accordingly, the complexes are colored in grey, blue, and red if there is an equal, higher or lower number of changing markers in the wild type compared to the mutant cells. **F)** Differential abundance analysis of all acetylated peptides between the Gcn5 catalytic dead mutant and the wild type. **G)** Signal intensities of Ada3 acetylated lysine residues 8 & 14 (left plot) and 182 (right plot) in wild type and Gcn5 catalytic dead cells under HU-stress and control conditions. **H)** Raw MS2 signal traces of Ada3 acetylated lysine residues 8 & 14 and 182 in the wild type and Gcn5 catalytic dead cells under HU-stress and control conditions. Note that the b10++ ion was not used for identification as it does not perfectly co-elute with the other peptide fragments. This could be caused by co-eluting small contaminant molecules which are small and in the same m/z range as the doubly charged b ion.
