# Supplementary material for: Global profiling of protein complex dynamics with an experimental library of protein interaction markers
Source: Nat Biotechnol. 2024 Oct 16;43(9):1562–76. doi: 10.1038/s41587-024-02432-8 (PMC12440823; doi:10.1038/s41587-024-02432-8)
Supplement: Supplementary file 2 — Reporting Summary [file 41587_2024_2432_MOESM2_ESM.pdf]

nature portfolio

Corresponding author(s): Paola Picotti

Last updated by author(s): 2024/09/05

Reporting Summary

Nature Portfolio wishes to improve the reproducibility of the work that we publish. This form provides structure for consistency and transparency in reporting. For further information on Nature Portfolio policies, see our Editorial Policies and the Editorial Policy Checklist.

Statistics

For all statistical analyses, confirm that the following items are present in the figure legend, table legend, main text, or Methods section.

n/a	Confirmed	
☐	☒	The exact sample size (*n*) for each experimental group/condition, given as a discrete number and unit of measurement
☐	☒	A statement on whether measurements were taken from distinct samples or whether the same sample was measured repeatedly
☐	☒	The statistical test(s) used AND whether they are one- or two-sided<br>*Only common tests should be described solely by name; describe more complex techniques in the Methods section.*
☐	☒	A description of all covariates tested
☐	☒	A description of any assumptions or corrections, such as tests of normality and adjustment for multiple comparisons
☐	☒	A full description of the statistical parameters including central tendency (e.g. means) or other basic estimates (e.g. regression coefficient) AND variation (e.g. standard deviation) or associated estimates of uncertainty (e.g. confidence intervals)
☐	☒	For null hypothesis testing, the test statistic (e.g. *F*, *t*, *r*) with confidence intervals, effect sizes, degrees of freedom and *P* value noted<br>*Give P values as exact values whenever suitable.*
☒	☐	For Bayesian analysis, information on the choice of priors and Markov chain Monte Carlo settings
☒	☐	For hierarchical and complex designs, identification of the appropriate level for tests and full reporting of outcomes
☒	☐	Estimates of effect sizes (e.g. Cohen's *d*, Pearson's *r*), indicating how they were calculated

Our web collection on statistics for biologists contains articles on many of the points above.

Software and code

Policy information about availability of computer code

Data collection	Mass-spectromety data was acquired using Xcalibur version 4.2 and version 4.3.
Data analysis	Raw mass-spectromety data was extracted using: Spectronaut version 15.6.211220.50606, Spectronaut version 17.1.221229.55965, Spectromine version 3.2.22022.52329, Skyline 64-bit (21.1)<br>Data analysis was performed in python version 3.7 and R version 4.1.1.<br>All code for data analysis is available on GitHub: https://github.com/cmarulli/FLiP-MS

For manuscripts utilizing custom algorithms or software that are central to the research but not yet described in published literature, software must be made available to editors and reviewers. We strongly encourage code deposition in a community repository (e.g. GitHub). See the Nature Portfolio guidelines for submitting code & software for further information.

Data

Policy information about availability of data

All manuscripts must include a data availability statement. This statement should provide the following information, where applicable:

- Accession codes, unique identifiers, or web links for publicly available datasets
- A description of any restrictions on data availability
- For clinical datasets or third party data, please ensure that the statement adheres to our policy

The mass spectrometry proteomics data have been deposited to the ProteomeXchange Consortium via the PRIDE partner repository with the dataset identifier

PXD055566.
PDB structures used in this study are available on https://www.rcsb.org. Accession codes of specific PDB structures highlighted in the publication are: 4DSS, 1JK0, 3QUW, 6OPC, 4C8Q, 6FAI

Human research participants

Policy information about studies involving human research participants and Sex and Gender in Research.

Reporting on sex and gender	Does not apply.
Population characteristics	Does not apply.
Recruitment	Does not apply.
Ethics oversight	Does not apply.

Note that full information on the approval of the study protocol must also be provided in the manuscript.

Field-specific reporting

Please select the one below that is the best fit for your research. If you are not sure, read the appropriate sections before making your selection.

☒ Life sciences ☐ Behavioural & social sciences ☐ Ecological, evolutionary & environmental sciences

For a reference copy of the document with all sections, see nature.com/documents/nr-reporting-summary-flat.pdf

Life sciences study design

All studies must disclose on these points even when the disclosure is negative.

Sample size	Our study uses data-independent acquisition mass-spectrometry and quantifies all detected proteins across the proteome, both in the generation of the FLiP library in yeast and in the subsequent comparison of wild type and mutant yeast in the presence and absence of hydroxyurea. In LiP-MS experiments, four replicates is standard practice. We measured four technical replicates for the FLiP-MS library and four biological replicates per condition for all other LiP-MS experiments (more details below in the replication section).
Data exclusions	To ensure that we only consider peptides reproducibly identified between biological replicates, we excluded all peptides that were not measured in at least triplicates in at least two conditions. We also excluded peptides belonging to proteins which could not be quantified in at least triplicates in at least two conditions.
Replication	All experiments were performed in quadruplicates. For generation of the FLiP library we used a single yeast culture to reduce biological variance, and then processed samples in 4 technical replicates. We measured four biological replicates per condition for all LiP-MS experiments comparing HU treatment and control in wild type and mutant yeast; each replicate used an independently generated yeast culture. We required a peptide to be measured in at least biological triplicates to be considered for further analysis. This was chosen to ensure high confidence in the data while not unduly penalizing low-abundance peptides. We include information about the number of replicates in all downstream statistical analysis.<br>The median coefficients of variation of all peptides of a given condition are below 22 % (with the exception of one with 28.1 %), showing good reproducibility. Hierarchical clustering at the peptide and protein level also confirmed that replicates of the same condition cluster together.
Randomization	The samples were randomized prior to LiP-MS processing. The randomized order was kept for the rest of the protocol including MS data acquisition.
Blinding	The randomized samples were only labeled with numbers. Different investigators performed randomization and limited proteolysis as well as the tryptic digest to ensure blinding. After data collection, samples were again assigned their label. Hierarchical clustering was performed to verify that replicates of the same condition cluster together.

Reporting for specific materials, systems and methods

We require information from authors about some types of materials, experimental systems and methods used in many studies. Here, indicate whether each material, system or method listed is relevant to your study. If you are not sure if a list item applies to your research, read the appropriate section before selecting a response.

Materials & experimental systems

n/a	Involved in the study
☐	☒ Antibodies
☒	☐ Eukaryotic cell lines
☒	☐ Palaeontology and archaeology
☐	☒ Animals and other organisms
☒	☐ Clinical data
☒	☐ Dual use research of concern

Methods

n/a	Involved in the study
☒	☐ ChIP-seq
☒	☐ Flow cytometry
☒	☐ MRI-based neuroimaging

Antibodies

Antibodies used	Primary antibody - Anti-GFP from mouse IgG1K (clones 7.1 and 13.1) - Roche SKU:11814460001<br>Secondary antibody - Goat anti-Mouse IgG (H+L) - Highly Cross-Adsorbed Secondary Antibody, HRP - Invitrogen Catalog # A16078<br>Primary antibody - Anti-FLAG from mouse IgG1 (clone M2) - ANTI-FLAG® M2 Affinity Gel - Millipore A2220
Validation	Primary antibody - Roche SKU:11814460001 - Validation statement on manufacturer's website: "Anti-GFP is tested for functionality and purity relative to a reference standard to confirm the quality of each new reagent preparation." "Monoclonal antibodies were further screened for performance in western blot and immunoprecipitation applications using GFP fusion proteins."<br><br>Secondary antibody - Highly Cross-Adsorbed Secondary Antibody, HRP - Invitrogen Catalog # A16078 - Validation statement on manufacturer's website: antibody was tested by chemiluminescent western blots.<br><br>Primary antibody - ANTI-FLAG® M2 Affinity Gel - Millipore A2220: Validation statement on manufacturer's website: was tested to have a minimal resin binding capacity of 0.6 mg/ml.

Animals and other research organisms

Policy information about studies involving animals; ARRIVE guidelines recommended for reporting animal research, and Sex and Gender in Research

Laboratory animals	We used the following S. cerevisae strains:<br>BY4716<br>BY4741 his3Δ1 ura3Δ0 leu2Δ0, met15Δ0<br>BY4741 gcn5-E173A his3Δ200 ura3-52 leu2Δ0 lys2-801 ade2-101 trp1Δ63<br>BY4741 MATa his3Δ1 leu2Δ0 ura3Δ0 met15Δ0 ada3-3xFLAG:kanMX6<br>BY4741 MATa his3Δ1 leu2Δ0 ura3Δ0 met15Δ0 spt7-GFP:HIS3
Wild animals	Does not apply.
Reporting on sex	Does not apply.
Field-collected samples	Does not apply.
Ethics oversight	The study used yeast. No ethical approval was needed.

Note that full information on the approval of the study protocol must also be provided in the manuscript.
