# Supplementary figures and images for: Global profiling of protein complex dynamics with an experimental library of protein interaction markers
Source: Nat Biotechnol. 2024 Oct 16;43(9):1562–76. doi: 10.1038/s41587-024-02432-8 (PMC12440823; doi:10.1038/s41587-024-02432-8)

CTR  
HU

250 -  
150 -  
100 -  
75 -  
55 -  
35 -  
25 -  
15 -

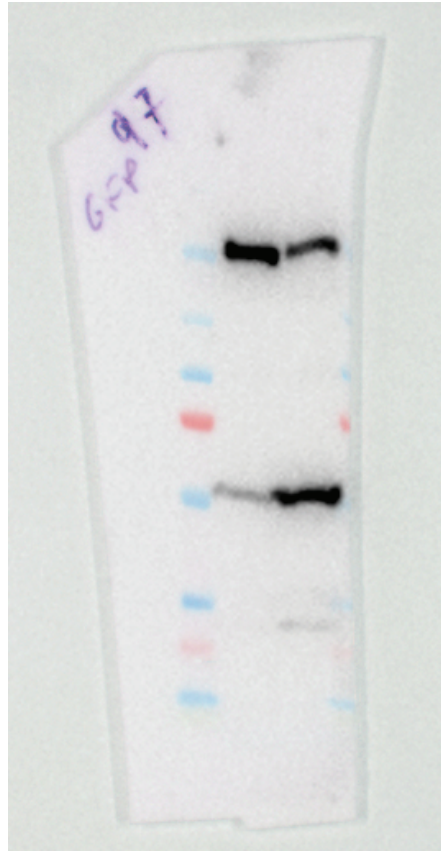

Supplement: Supplementary file 4 — Unprocessed western blot. [file 41587_2024_2432_MOESM4_ESM.pdf]
